# A Density of Ramified Primes

**DOI:** 10.1007/s40993-021-00295-5

**Published:** 2021-11-15

**Authors:** Stephanie Chan, Christine McMeekin, Djordjo Milovic

**Affiliations:** 1 Department of Mathematics, University College London, London, UK; 2 Max-Planck-Institut für Mathematik, Bonn, Germany

**Keywords:** Density, Ramified Primes, Spin, Distribution, 11R45, 11N05, 11N36, 11R80, 11S15, 11L20, 11L40

## Abstract

Let *K* be a cyclic number field of odd degree over ${\mathbb {Q}}$ with odd narrow class number, such that 2 is inert in $K/{\mathbb {Q}}$. We define a family of number fields $\{K(p)\}_p$, depending on *K* and indexed by the rational primes *p* that split completely in $K/{\mathbb {Q}}$, in which *p* is always ramified of degree 2. Conditional on a standard conjecture on short character sums, the density of such rational primes *p* that exhibit one of two possible ramified factorizations in $K(p)/{\mathbb {Q}}$ is strictly between 0 and 1 and is given explicitly as a formula in terms of the degree of the extension $K/{\mathbb {Q}}$. Our results are unconditional in the cubic case. Our proof relies on a detailed study of the joint distribution of spins of prime ideals.

## Introduction

Given a number field *K*, let ${\mathcal {O}}$, $\mathrm {Cl}$, and $\mathrm {Cl}^+$ denote its ring of integers, its class group, and its narrow class group, respectively. We will prove certain density theorems for number fields *K* satisfying the following conditions:

1. $K/{\mathbb {Q}}$ is Galois with cyclic Galois group;
2. $$[K:{\mathbb {Q}}]>1$$ is odd;
3. $$h^+ =|\mathrm {Cl}^+|$$ is odd;
4. the prime 2 is inert in $K/{\mathbb {Q}}$.

Recall that $\mathrm {Cl}^+$ is the quotient of the group of invertible fractional ideals of *K* by the subgroup of principal fractional ideals that can be generated by a totally positive element; in other words, $\mathrm {Cl}^+$ is the ray class group of conductor equal to the product of all real places. If $\alpha \in K$ is totally positive, i.e., if $\sigma (\alpha )>0$ for all real embeddings $\sigma :K\hookrightarrow {\mathbb {R}}$, we will sometimes write $\alpha \succ 0$. If $h^+$ is odd for a totally real field, then

1 $$\begin{aligned} {\mathcal {O}}^{\times }_{+} := \{u\in {\mathcal {O}}^{\times }: u\succ 0\} = \left( {\mathcal {O}}^{\times }\right) ^2. \end{aligned}$$

Notice that if $K/{\mathbb {Q}}$ is an odd degree Galois extension, then $K/{\mathbb {Q}}$ is totally real. Since $[\mathrm {Cl}^+:\mathrm {Cl}]$ is always a power of 2, the condition (C3) implies that $\mathrm {Cl}^+= \mathrm {Cl}$. Therefore the conditions that $K/{\mathbb {Q}}$ is Galois, satisfying (C2) and (C3) together imply the following:

1. $K/{\mathbb {Q}}$ is Galois, *K* is totally real, and $\mathrm {Cl}^+ = \mathrm {Cl}$.

If *K* is totally real, then $\mathrm {Cl}^+ = \mathrm {Cl}$ if and only if every totally positive unit in ${\mathcal {O}}$ is a square; see Lemma 1. Hence, property (P1) can be restated as

1. $K/{\mathbb {Q}}$ is Galois, *K* is totally real, and ${\mathcal {O}}^{\times }_{+} := \{u\in {\mathcal {O}}^{\times }: u\succ 0\} = \left( {\mathcal {O}}^{\times }\right) ^2$.

Number fields satisfying property (C1) and (P1) were studied by Friedlander, Iwaniec, Mazur, and Rubin [5]. More precisely, Friedlander et al. proved that if $\sigma$ is a (fixed) generator of ${{\,\mathrm{Gal}\,}}(K/{\mathbb {Q}})$, then the density of principal prime ideals $\pi {\mathcal {O}}$ that split in the quadratic extension $K(\sqrt{\sigma (\pi )})/K$ is equal to 1/2. Koymans and Milovic [6] extended the results of Friedlander et al. in two different aspects. First, the number field *K* now needs to satisfy only property (P1), i.e., $K/{\mathbb {Q}}$ need not be cyclic; second, density theorems about the splitting behavior of principal prime ideals are proved for multi-quadratic extensions of the form $K(\{\sqrt{\sigma (\pi )}: \sigma \in \Sigma \})/K$, where $\Sigma$ is a fixed subset of ${{\,\mathrm{Gal}\,}}(K/{\mathbb {Q}})$ with the property that $\sigma \not \in \Sigma$ whenever $\sigma ^{-1}\in \Sigma$.

Our main goal is to further extend these results to a certain setting where $\Sigma = {{\,\mathrm{Gal}\,}}(K/{\mathbb {Q}})\setminus \{1\}$; in this setting, we in fact have $\sigma \in \Sigma$ whenever $\sigma ^{-1}\in \Sigma$, and so our work features a new interplay of the Chebotarev Density Theorem and the method of sums of type I and type II. In particular, the densities appearing in our main theorems are of greater complexity than those appearing in [5] or [6].

Another innovation in our work is that by assuming property (C3), we are now also able to study the splitting behavior of *all* prime ideals, and not only those that are principal. While our generalization of “spin” to non-principal ideals may appear innocuous (see Definition 3), it is of note that it still encodes the relevant splitting information as well as that the study of its oscillations requires new ideas, carried out in Sect. 6.

Let *K* be a number field satisfying properties (P1) and (C3), and let *p* be a rational prime that splits completely in $K/{\mathbb {Q}}$. We will now define an extension $K(p)/{\mathbb {Q}}$ where *p* ramifies; this extension was first studied by McMeekin [8]. Let ${\mathfrak {p}}$ be an unramified prime ideal of degree one in ${\mathcal {O}}$. Let $R_{{\mathfrak {p}}}^{+}$ denote the maximal abelian extension of *K* unramified at all finite primes other than ${\mathfrak {p}}$; in other words, $R_{{\mathfrak {p}}}^{+}$ is the ray class field of *K* of conductor ${\mathfrak {p}}\infty$, where $\infty$ denotes the product of all real places of *K*. There is a unique subfield $K({\mathfrak {p}})\subset R_{{\mathfrak {p}}}^{+}$ of degree 2 over *K* when ${\mathfrak {p}}$ is prime to 2 (see Lemma 2). Finally, we define *K*(*p*) to be the compositum of $K({\mathfrak {p}})$ over all primes ${\mathfrak {p}}$ lying above *p*, i.e.,

$$\begin{aligned} K(p) = \prod _{{\mathfrak {p}}|p}K({\mathfrak {p}}). \end{aligned}$$

As $K(p)/{\mathbb {Q}}$ is Galois, the residue field degree $f_{K(p)/{\mathbb {Q}}}(p)$ of *p* in $K(p)/{\mathbb {Q}}$ is well-defined. Our goal is to study the distribution of $f_{K(p)/{\mathbb {Q}}}(p)$ as *p* varies. Note that because *p* splits completely in $K/{\mathbb {Q}}$, $f_{K(p)/{\mathbb {Q}}}(p)$ is equal to the residue field degree $f_{K(p)/K}({\mathfrak {p}})$ of ${\mathfrak {p}}$ in $K(p)/{\mathbb {Q}}$ for any prime ${\mathfrak {p}}$ of *K* lying above *p*. Furthermore, $f_{K(p)/{\mathbb {Q}}}(p)=f_{K(p)/K}({\mathfrak {p}})$ must divide 2 since [*K*(*p*) : *K*] is a power of 2 and there are no cyclic subgroups of ${{\,\mathrm{Gal}\,}}(K(p)/K)$ of order greater than 2.

To state our main results, we now introduce the relevant notions of density. For sets of primes $A\subseteq B$, we define the density of *A* restricted to *B* to be

$$\begin{aligned} d(A|B):= \lim _{N\rightarrow \infty } \frac{\#A|_N}{\#B|_N}. \end{aligned}$$

where $A|_N:=\{p \in A: {\mathfrak {N}}(p)<N\}$ and $B|_N$ is defined similarly. When $\Pi$ consists of all but finitely many primes, then $d(A):=d(A|\Pi )$ is the usual natural density of *A*. (The notation *d*(*A*|*B*) is chosen to highlight an analogy to conditional probability.)

Let ${\mathscr {P}}_{\mathbb {Q}}^{2}$ denote the set of rational primes co-prime to 2. For a fixed sign, $\mu \in \{\pm \}$, we define the following sets of rational primes.

$$\begin{aligned} \begin{array}{ l l} S &{} {:}{=} \{p \in {\mathscr {P}}_{\mathbb {Q}}^{2}: p \text { splits completely in } K/{\mathbb {Q}}\}, \\ S_\mu &{} {:}{=}\{p\in S: p \equiv \mu 1 \bmod 4{\mathbb {Z}}\}, \\ F &{} {:}{=} \{ p \in S: f_{K(p)/{\mathbb {Q}}}(p) = 1\}, \\ F_\mu &{} {:}{=} S_\mu \cap F. \end{array} \end{aligned}$$

Our main results are conditional on the following conjecture, a slight variant of which appears in both [5] and [6]. In the following conjecture, the real number $\eta \in (0, 1]$ plays the role of 1/*n* from [5, Conjecture $C_n$, p. 738-739].

### Conjecture 1

($C_{\eta }$ [5]) Let $\eta$ be a real number satisfying $0<\eta \le 1$. Then there exists a real number $\delta =\delta (\eta )>0$ such that for all $\epsilon >0$ there exists a real number $C=C(\eta , \epsilon )>0$ such that for all integers $Q\ge 3$, all real non-principal characters $\chi$ of conductor $q\le Q$, all integers $N\le Q^{\eta }$, and all integers *M*, we have

$$\begin{aligned} \left| \sum _{M<a\le M+N}\chi (a)\right| \le C Q^{\eta (1-\delta )+\epsilon }. \end{aligned}$$

We note that Conjecture $C_{\eta }$ is known for $\eta >1/4$, as a consequence of the classical Burgess’s inequality [2], and remains open for $\eta \le 1/4$. Moreover, for sums as above starting at $M = 0$, Conjecture $C_{\eta }$ (for any $\eta$) is a consequence of the Grand Riemann Hypothesis for the *L*-function $L(s, \chi )$. We are now ready to state our main results.

### Theorem 1

Let *K* be a number field of degree *n* satisfying conditions (C1)-(C4). Assume Conjecture $C_{\eta }$ holds for $\eta = \frac{2}{n(n-1)}$. For $k\ne 1$ dividing *n* let $d_k$ be the order of 2 in $({\mathbb {Z}}/k)^\times$. Then for a fixed sign $\mu \in \{\pm \}$,

$$\begin{aligned} d(F_\mu |S_\mu )=\frac{s_\mu }{2^{3(n-1)/2}}, \quad \text {and} \quad d(F|S) = \frac{s_++s_-}{2^{(3n-1)/2}} \end{aligned}$$

where

$$\begin{aligned}s_+= \prod _{\begin{array}{c} k\mid n \\ d_k\text {odd}\\ k\ne 1 \end{array}}(2^{1+d_k}-1)^{\frac{\phi (k)}{2d_k}},\end{aligned}$$

and

$$\begin{aligned}s_-=\prod _{\begin{array}{c} k\mid n \\ d_k\text {even}\\ k\ne 1 \end{array}}(2^{d_k/2}+1)^{\frac{\phi (k)}{d_k}}\prod _{\begin{array}{c} k\mid n \\ d_k\text {odd}\\ k\ne 1 \end{array}}(2^{d_k}-1)^{\frac{\phi (k)}{2d_k}},\end{aligned}$$

where $\phi$ denotes the Euler’s totient function. In particular, when *n* is prime, writing $d=d_n$,

$$\begin{aligned} (s_+,s_-) ={\left\{ \begin{array}{ll} \left( (2^{1+d}-1)^{\frac{n-1}{2d}},\ (2^d-1)^{\frac{n-1}{2d}}\right) &{} \text { if }d\text { is odd,}\\ \quad \left( 1,\ (2^{\frac{d}{2}}+1)^{\frac{n-1}{d}}\right) &{} \text { if }d\text { is even.} \end{array}\right. } \end{aligned}$$

The density *d*(*F*|*S*) is determined by the product of densities *d*(*F*|*R*) and *d*(*R*|*S*) where *R* is the set of primes satisfying a certain Hilbert symbol condition. Toward computing the density *d*(*R*|*S*), the terms $s_\mu$ arise from counting the number of solutions to this Hilbert symbol condition over $({\mathcal {O}}/4)^\times /(({\mathcal {O}}/4)^\times )^2$.

**Table 1:** Densities from Theorem 1, computed for *K* of degree *n* satisfying the necessary hypotheses

*n*	$d(F_+|S_+)$	$d(F_-|S_-)$	*d*(*F*|*S*)
3	1/8	3/8	1/4
5	1/64	5/64	3/64
7	15/512	7/512	11/512
9	1/4096	27/4096	7/2048
11	1/32768	33/32768	17/32768
13	1/262144	65/262144	33/262144
15	1/2097152	375/2097152	47/262144

In the cubic case, we have the following unconditional theorem.

### Theorem 2

Let $K/{\mathbb {Q}}$ be a cubic cyclic number field and odd class number in which 2 is inert. Then

$$\begin{aligned} d(F|S)=\frac{1}{4}, \end{aligned}$$

$$\begin{aligned} d(F_+|S_+) = \frac{1}{8}, \quad \text {and} \quad d(F_-|S_-) = \frac{3}{8}. \end{aligned}$$

For our main results, we have assumed that *K* satisfies properties (C1)-(C4). To start, we need properties (P1) and (C3) to define the extensions *K*(*p*)/*K* for primes *p* that split completely in $K/{\mathbb {Q}}$. Coincidentally, as mentioned above, property (C3) also allows us to study the splitting behavior of all (not necessarily principal) prime ideals. Property (C2) ensures that ${{\,\mathrm{Gal}\,}}(K/{\mathbb {Q}})$ contains no involutions. While methods to deal with involutions do exist (see [5, Section 12, p. 745]), incorporating them into our arguments is non-trivial and may pose interesting new challenges in our analytic arguments. Properties (C1) and (C4) simplify our combinatorial arguments and allow us to give explicit density formulas. Removing the assumptions of properties (C1) and (C4) would pose new combinatorial challenges.

To end this section, we give some examples of number fields satisfying (C1)-(C4) so as to convince the reader that our theorems are not vacuous. First, many such fields can be found within the parametric families given by Friedlander et al. in [5, p. 712] and originally due to Shanks [14] and Lehmer [7], namely

$$\begin{aligned} \{{\mathbb {Q}}(\alpha _m):\ m\in {\mathbb {Z}}\}\quad \text {and}\quad \{{\mathbb {Q}}(\beta _m):\ m\in {\mathbb {Z}}\} \end{aligned}$$

where $\alpha _m$ and $\beta _m$ are roots of the polynomials

$$\begin{aligned} f_m(x) = x^3 + m x^2 + (m - 3) x - 1. \end{aligned}$$

and

$$\begin{aligned} g_m(x)= & {} x^5+m^2x^4-2(m^3+3m^2+5m+5)x^3 \\&+(m^4+5m^3+11m^2+15m+5)x^2+(m^3+4m^2+10m+10)x+1, \end{aligned}$$

respectively. Such fields always satisfy properties (P1), (C1), and (C2). We also note that one can use the law of cubic reciprocity to show that the fields ${\mathbb {Q}}(\alpha _m)$ always satisfy property (C4). For small *m* one can check the remaining properties using Sage or another similar mathematical software package. For instance, if $\beta _7$ is any root of

$$\begin{aligned} g_7(x) = x^5 + 49x^4 - 1060x^3 + 4765x^2 + 619x + 1, \end{aligned}$$

then ${\mathbb {Q}}(\beta _7)$ is a totally real cyclic degree-5 number field of class number 1451 where 2 stays inert.

More generally, we can look for special subfields of cyclotomic fields. Let *m* be a prime number and $\zeta _{m}$ a primitive *m*-th root of unity, so that ${\mathbb {Q}}(\zeta _{m})/{\mathbb {Q}}$ is a cyclic extension of degree $\varphi (m)$, and suppose that *n* is an odd integer such that $\varphi (m)\equiv 0\bmod 2n$. For instance, we can take *n* to be a Sophie Germain prime and then take $m = 2n+1$ to also be a prime. Suppose also that 2 is inert in ${\mathbb {Q}}(\zeta _{m})$, i.e., that 2 is a primitive root modulo *m*. We then define *K* to be the unique subfield of ${\mathbb {Q}}(\zeta _{m}+\zeta _{m}^{-1})$ of degree *n* over ${\mathbb {Q}}$; *K* readily satisfies properties (C1), (C2), (C4), while for small *n* the property that $\mathrm {Cl}^+ = \mathrm {Cl}$ and property (C3) can be checked using Sage. For instance, the unique degree-5 subfield of ${\mathbb {Q}}(\zeta _{191})$ has class number 11; it is isomorphic to ${\mathbb {Q}}(\beta _2)$ with $\beta _2$ a root of the polynomial $g_2$ as above.

## Two families of number fields

We say a modulus ${\mathfrak {m}}$ is *narrow* whenever it is divisible by all real infinite places. We say a modulus is *wide* whenever it is not divisible by any infinite place. We say a ray class group or ray class field is narrow or wide whenever its conductor is narrow or wide respectively.

For ${\mathfrak {m}}$ an ideal of ${\mathcal {O}}$, let $\mathrm {Cl}^+_{\mathfrak {m}}$ denote the narrow ray class group of conductor ${\mathfrak {m}}$. That is, $\mathrm {Cl}^+_{\mathfrak {m}}$ is the ray class group with conductor divisible by all real infinite places with finite part ${\mathfrak {m}}$.

The following lemma leads to several equivalent formulations of property (P1).

### Lemma 1

*K* is any number field.

1. The following are equivalent.
  - $$\mathrm {Cl}^+=\mathrm {Cl}$$.
  - Every principal ideal has a totally positive generator.
  - All signatures are represented by units.
2. If $h^+$ is odd, then $\mathrm {Cl}^+=\mathrm {Cl}$.
3. *K* is totally real with $\mathrm {Cl}^+=\mathrm {Cl}$ if and only if ${\mathcal {O}}^{\times }_{+} = \left( {\mathcal {O}}^{\times }\right) ^2$.

Here, if *K* is not necessarily totally real, an element is said to be totally positive when it is positive in all real embeddings, and the signature of an element is determined by the signs of the element in each real embedding.

### Proof

Let *K* be an arbitrary number field with $r_1$ real embeddings and $r_2$ pairs of complex embeddings. That (a) and (b) are equivalent follows from the definitions of the narrow and wide Hilbert class fields. By the exact sequence and canonical isomorphism in [10, Theorem V.1.7] applied to the narrow modulus with trivial finite part, condition (a) is true exactly when ${\mathcal {O}}^\times /{\mathcal {O}}^\times _+ \cong ({\mathbb {Z}}/2)^{r_1}$. Noting that there are $r_1$ signatures and the signatures of two units are equal exactly when these units are equivalent modulo the totally positive units, (a) is equivalent to (c). Since $[\mathrm {Cl}^+:\mathrm {Cl}]$ is always a power of 2, if $h^+$ is odd, then property (a) holds.

As noted above, condition (a) is true exactly when ${\mathcal {O}}^\times /{\mathcal {O}}^\times _+ \cong ({\mathbb {Z}}/2)^{r_1}$. By Dirichlet’s unit theorem, ${\mathcal {O}}^\times /({\mathcal {O}}^\times )^2\cong \left( {\mathbb {Z}}/2\right) ^{r_1+r_2}$. Therefore if (a) holds and in addition *K* is totally real, then $r_2=0$ and ${\mathcal {O}}^\times /{\mathcal {O}}^\times _+ \cong {\mathcal {O}}^\times /({\mathcal {O}}^\times )^2$. Containment of $({\mathcal {O}}^\times )^2$ in ${\mathcal {O}}^\times _+$ gives equality. Conversely, if we assume ${\mathcal {O}}^{\times }_{+} = \left( {\mathcal {O}}^{\times }\right) ^2$, then ${\mathcal {O}}^\times /{\mathcal {O}}^\times _+ \cong \left( {\mathbb {Z}}/2\right) ^{r_1+r_2}$ by Dirichlet’s unit theorem. By the exact sequence and canonical isomorphism in [10, Theorem V.1.7], there is an injection from ${\mathcal {O}}^\times /{\mathcal {O}}^\times _+$ into a group isomorphic to $\left( {\mathbb {Z}}/2\right) ^{r_1}$. Therefore $r_2=0$ and ${\mathcal {O}}^\times /{\mathcal {O}}^\times _+ \cong \left( {\mathbb {Z}}/2\right) ^{r_1}$. That is, *K* is totally real and condition (a) holds. $\square$

### Lemma 2

Let *K* be a totally real number field with odd narrow class number $h^+$. Let ${\mathfrak {p}}$ be an odd prime of *K*. Then the narrow ray class field over *K* of conductor ${\mathfrak {p}}$ has a unique subextension that is quadratic over *K*.

### Proof

Let ${\mathcal {O}}^\times _{{\mathfrak {p}},1}$ denote the totally positive units of *K* that are congruent to 1 modulo ${\mathfrak {p}}$. The exact sequence from class field theory as in [10, V.1.7] induces the following short exact sequence on the $2-$torsion subgroups, where surjectivity of the final map is due to the assumption that $h^+$ is odd.

$$\begin{aligned} 1 \rightarrow {\mathcal {O}}^\times /{\mathcal {O}}_{{\mathfrak {p}},1}^\times [2^\infty ] \rightarrow ({\mathbb {Z}}/2)^n \times ({\mathcal {O}}/{\mathfrak {p}})^\times [2^\infty ] \rightarrow \mathrm {Cl}_{\mathfrak {p}}^+[2^\infty ] \rightarrow 1. \end{aligned}$$

By Lemma 1, all signatures are represented by units. Letting $\#({\mathcal {O}}^\times /{\mathcal {O}}_{{\mathfrak {p}},1}^\times [2^\infty ] ) = m2^k$ for odd *m*, for *u* any unit, $u^m \in {\mathcal {O}}^\times /{\mathcal {O}}_{{\mathfrak {p}},1}^\times [2^\infty ]$ and $u^m$ shares the same signature as *u*. Therefore the first map is surjective onto the projection to $({\mathbb {Z}}/2)^n$. Since $\mathrm {Cl}_{\mathfrak {p}}^+[2^\infty ]$ is isomorphic to the quotient of $({\mathbb {Z}}/2)^n \times ({\mathcal {O}}/{\mathfrak {p}})^\times [2^\infty ]$ by ${\mathcal {O}}^\times /{\mathcal {O}}_{{\mathfrak {p}},1}^\times [2^\infty ]$, this shows that $\mathrm {Cl}_{\mathfrak {p}}^+[2^\infty ]$ is cyclic.

The first map in this short exact sequence is not surjective because any element of $({\mathbb {Z}}/2)^n \times ({\mathcal {O}}/{\mathfrak {p}})^\times [2^\infty ]$ of the form (0, *x*) must come from a square because ${\mathcal {O}}^\times _+ = ({\mathcal {O}}^\times )^2$ by Lemma 1. Then the size of $\mathrm {Cl}_{\mathfrak {p}}^+[2^\infty ]$ is nontrivial, so since these are all 2-groups $\#\mathrm {Cl}_{\mathfrak {p}}^+[2^\infty ]$ is even. $\square$

We may now define the multi-quadratic extension *K*(*p*)/*K* as in Sect. 1. In addition, we define another family of number fields parameterized by prime numbers *p* for which our results also hold. For both families of number fields, we consider a totally real number field *K* with odd narrow class number $h^+$. Furthermore, we now impose the condition that $K/{\mathbb {Q}}$ is a Galois extension. Equivalently, we are assuming conditions (P1) and (C3).

In Definition 1, we apply Lemma 2 to ensure the existence of a unique quadratic subextension of the narrow ray class field over *K* of conductor ${\mathfrak {p}}$. In Definition 2 we will use the fact that for such *K*, a principal ideal always has a totally positive generator; see Lemma 1.

### Definition 1

Given an odd rational prime *p* that splits completely in $K/{\mathbb {Q}}$ and a prime ideal ${\mathfrak {p}}\subset {\mathcal {O}}$ lying above *p*, define $K({\mathfrak {p}})$ to be the unique quadratic subextension of the narrow ray class field over *K* of conductor ${\mathfrak {p}}$.

Define *K*(*p*) to be the compositum of the fields $K({\mathfrak {p}}^{\sigma })$ as $\sigma$ ranges over ${{\,\mathrm{Gal}\,}}(K/{\mathbb {Q}})$.

### Definition 2

Given an odd rational prime *p* that splits completely in $K/{\mathbb {Q}}$, a prime ideal ${\mathfrak {p}}\subset {\mathcal {O}}$ lying above *p*, and a totally positive generator $\alpha$ of the principal ideal ${\mathfrak {p}}^h$, we define

$$\begin{aligned} K_+({\mathfrak {p}}){:}{=} K(\sqrt{\alpha }).\end{aligned}$$

Define $K_+(p)$ to be the compositum of the number fields $K_+({\mathfrak {p}}^{\sigma })$ as $\sigma$ ranges over ${{\,\mathrm{Gal}\,}}(K/{\mathbb {Q}})$.

Since *K* is totally real and $h_+$ is odd, Lemma 1 implies that ${\mathcal {O}}^{\times }_+ = ({\mathcal {O}}^{\times })^2$, so $K_+({\mathfrak {p}})$ does not depend on the choice of totally positive generator $\alpha$.

We note that while each of the fields $K({\mathfrak {p}}^{\sigma })$ need not be Galois over ${\mathbb {Q}}$, their compositum *K*(*p*) certainly is. Similarly, $K_+(p)/{\mathbb {Q}}$ is Galois, and each of the extensions $K_+({\mathfrak {p}}^{\sigma })/{\mathbb {Q}}$ need not be.

For an abelian extension of number fields *L*/*E* and a prime ${\mathfrak {p}}$ of *E*, let $f_{L/E}({\mathfrak {p}})$ denote the residue field degree of ${\mathfrak {p}}$ in *L*/*E* and $e_{L/E}({\mathfrak {p}})$ the ramification index of ${\mathfrak {p}}$ in *L*/*E*. In particular, the ramification indices and residue field degrees $e_{K(p)/{\mathbb {Q}}}(p)$, $f_{K(p)/{\mathbb {Q}}}(p)$, $e_{K_+(p)/{\mathbb {Q}}}(p)$, and $f_{K_+(p)/{\mathbb {Q}}}(p)$ are well-defined.

Since *p* is assumed to split completely in $K/{\mathbb {Q}}$, there are *n* distinct primes in *K* lying above *p*, and they are of the form ${\mathfrak {p}}^{\sigma }$, where ${\mathfrak {p}}$ is one such prime and $\sigma$ ranges over ${{\,\mathrm{Gal}\,}}(K/{\mathbb {Q}})$.

By Lemma 2, $K({\mathfrak {p}}^\sigma )/K$ is a quadratic extension, and since $\alpha$ generates a prime ideal, $K_+({\mathfrak {p}}^\sigma )/K$ is also a quadratic extension. Since $K({\mathfrak {p}}^\sigma )$ is a subfield of the narrow ray class field over *K* of conductor ${\mathfrak {p}}^\sigma$, the extension $K({\mathfrak {p}}^\sigma )/K$ is unramified at ${\mathfrak {p}}^\tau$ for all $\tau \ne \sigma$ in ${{\,\mathrm{Gal}\,}}(K/{\mathbb {Q}})$. Since $h^+$ is odd, $K({\mathfrak {p}}^\sigma )/K$ is ramified at ${\mathfrak {p}}^\sigma$. Therefore $e_{K(p)/{\mathbb {Q}}}(p)=2$ and $[K(p):{\mathbb {Q}}]=n2^n$ where $n=[K:{\mathbb {Q}}]$.

Since ${\mathfrak {p}}^\sigma$ is an odd prime, ${\mathfrak {p}}^\sigma$ divides the discriminant of $K_+({\mathfrak {p}}^\sigma )/K$ and so this extension is ramified at ${\mathfrak {p}}^\sigma$. Since ${\mathfrak {p}}^\tau$ does not divide the discriminant for any $\tau \ne \sigma$ in ${{\,\mathrm{Gal}\,}}(K/{\mathbb {Q}})$, $K_+({\mathfrak {p}}^\sigma )/K$ is unramified at ${\mathfrak {p}}^\tau$ for all such $\tau$. Therefore $e_{K_+(p)/{\mathbb {Q}}}(p)=2$ and $[K_+(p):{\mathbb {Q}}]=n2^n$.

The residue field ${\mathbb {Z}}/p$ is cyclic and injects into ${\mathcal {O}}_{K(p)}/{\mathfrak {P}}$ where ${\mathfrak {P}}$ is a prime of *K*(*p*) above *p*. Therefore $f_{K(p)/{\mathbb {Q}}}(p) \mid 2$ because there are no cyclic subextensions of *K*(*p*)/*K* of degree greater than 2, and *p* is assumed to split completely in $K/{\mathbb {Q}}$. Similarly, $f_{K_+(p)/{\mathbb {Q}}}(p) \mid 2$. We summarise in the following Lemma.

### Lemma 3

Let *K* be a totally real number field of degree *n* that is Galois over ${\mathbb {Q}}$ with odd narrow class number and let *p* be a prime that splits completely in $K/{\mathbb {Q}}$. For $L=K(p)$ and for $L=K_+(p)$,

1. $L/{\mathbb {Q}}$ is a Galois extension of degree $n2^n$.
2. $$e_{L/{\mathbb {Q}}}(p) = 2$$.
3. $$f_{L/{\mathbb {Q}}}(p)\mid 2$$.

We will see in Corollary 1 that for a fixed odd rational prime *p* splitting completely in $K/{\mathbb {Q}}$, the residue field degrees of *p* in $K(p)/{\mathbb {Q}}$ and in $K_+(p)/{\mathbb {Q}}$ are equal. Hence, to prove Theorem 1, we will prove the analogous results for the family of extensions $K_+(p)/{\mathbb {Q}}$.

## The spin of prime ideals

Throughout this section, we will assume *K* satisfies (P1) and (C3). By Lemma 1, this is equivalent to assuming that *K* is a totally real number field that is Galois over ${\mathbb {Q}}$ with odd narrow class number, and these conditions imply that ${\mathcal {O}}^{\times }_{+} =\left( {\mathcal {O}}^{\times }\right) ^2$. We give the following definition of *spin*, which extends the definition of spin from [5, (1.1)] in a natural way so that it applies to all odd ideals (not necessary principal).

### Definition 3

Let $\sigma \in {{\,\mathrm{Gal}\,}}(K/{\mathbb {Q}})$ be non-trivial. Given an odd ideal ${\mathfrak {a}}$, we define the spin of ${\mathfrak {a}}$ (with respect to $\sigma$) to be

$$\begin{aligned} \text {spin}({\mathfrak {a}},\sigma ) = \left( \frac{\alpha }{ {\mathfrak {a}}^\sigma } \right) , \end{aligned}$$

where $\alpha$ is any totally positive generator of the principal ideal ${\mathfrak {a}}^h$, and where $\left( \frac{\cdot }{\cdot } \right)$ denotes the quadratic residue symbol in *K*.

The assumption ${\mathcal {O}}^{\times }_{+} =\left( {\mathcal {O}}^{\times }\right) ^2$ is important for two reasons. First, Lemma 1 ensures that the principal ideal ${\mathfrak {a}}^h$ has a generator $\alpha$ that is totally positive. Second, any two totally positive generators of ${\mathfrak {a}}^h$ differ by a square, so the value of the quadratic residue symbol defining the spin does not depend on the choice of totally positive generator $\alpha$.

If ${\mathfrak {a}}$ is an odd principal ideal and $\alpha _0$ is a totally positive generator of ${\mathfrak {a}}$, then $\alpha _0^h$ is a totally positive generator for ${\mathfrak {a}}^h$. As *h* is odd, we have

$$\begin{aligned} \left( \frac{\alpha _0}{ {\mathfrak {a}}^\sigma } \right) = \left( \frac{\alpha _0^h}{ {\mathfrak {a}}^\sigma } \right) , \end{aligned}$$

so our definition coincides with that of Friedlander et al. in [5] for odd principal ideals ${\mathfrak {a}}$.

### Known results

The main result in [5] can be stated as follows.

#### Theorem 3

([5, Theorem 1.1]) Suppose *K* is a number field satisfying properties (P1) and (C1). Suppose $n = [K:{\mathbb {Q}}]\ge 3$. Assume Conjecture $C_{\eta }$ holds for $\eta = 1/n$ with $\delta = \delta (\eta )>0$. Let $\sigma$ be a generator of the Galois group ${{\,\mathrm{Gal}\,}}(K/{\mathbb {Q}})$. Then for all real numbers $x>3$, we have

$$\begin{aligned} \sum _{\begin{array}{c} {\mathfrak {p}}\text { principal} \\ \text {prime ideal} \\ {\mathfrak {N}}({\mathfrak {p}}) \le x \end{array}} {{\,\mathrm{spin}\,}}({\mathfrak {p}},\sigma ) \ll x^{1-\theta +\epsilon } \end{aligned}$$

where $\theta =\theta (n)=\frac{\delta }{2n(12n+1)}$. Here the implied constant depends only on $\epsilon$ and *K*.

Friedlander et al. also proved an analogous result for the case when the summation is restricted to principal prime ideals ${\mathfrak {p}}$ with totally positive generators satisfying a suitable congruence condition.

By Burgess’s inequality, Conjecture $C_{\eta }$ holds for $\eta =1/3$ with $\delta = \frac{1}{48}$, so Theorem 3 holds unconditionally for $[K:{\mathbb {Q}}]=3$ where $\theta = \frac{1}{10656}$.

In [5, Section 11], Friedlander et al. pose some questions about the joint distribution of ${{\,\mathrm{spin}\,}}({\mathfrak {p}}, \sigma )$ and ${{\,\mathrm{spin}\,}}({\mathfrak {p}}, \tau )$ as ${\mathfrak {p}}$ varies over prime ideals, where $\sigma$ and $\tau$ are two distinct generators of the cyclic group ${{\,\mathrm{Gal}\,}}(K/{\mathbb {Q}})$. In [6], Koymans and Milovic prove that such spins are distributed independently if $n\ge 5$, i.e., that the product ${{\,\mathrm{spin}\,}}({\mathfrak {p}}, \sigma ){{\,\mathrm{spin}\,}}({\mathfrak {p}}, \tau )$ oscillates similarly as in Theorem 3. In fact, they prove that the product of spins

$$\begin{aligned} \prod _{\sigma \in S}{{\,\mathrm{spin}\,}}({\mathfrak {p}}, \sigma ) \end{aligned}$$

oscillates as long as the fixed non-empty subset *S* of ${{\,\mathrm{Gal}\,}}(K/{\mathbb {Q}})$ satisfies the property that $\sigma \not \in S$ whenever $\sigma ^{-1}\in S$. Moreover, their result holds for number fields *K* satisfying property (P1) and having arbitrary Galois groups, i.e., not necessarily satisfying property (C1).

The assumption in [6] that $\sigma \not \in S$ whenever $\sigma ^{-1}\in S$ is made because ${{\,\mathrm{spin}\,}}({\mathfrak {p}}, \sigma )$ and ${{\,\mathrm{spin}\,}}({\mathfrak {p}}, \sigma ^{-1})$ are not independent in the following sense. For a place *v* of *K*, let $K_{v}$ denote the completion of *K* at *v*. For $a,b\in K$ coprime to *v*, the Hilbert Symbol $(a, b)_v$ is defined to be 1 if the equation $ax^2+by^2=z^2$ has a solution $x,y,z\in K_{v}$ with at least one of *x*, *y*, or *z* non-zero and $-1$ otherwise.

#### Proposition 1

([5, Lemma 11.1]) Suppose *K* is a number field satisfying properties (P1) and (C3). Suppose ${\mathfrak {p}}\subset {\mathcal {O}}$ is a prime ideal and $\sigma \in {{\,\mathrm{Gal}\,}}(K/{\mathbb {Q}})$ is an automorphism such that ${\mathfrak {p}}$ and ${\mathfrak {p}}^\sigma$ are relatively prime. Then

$$\begin{aligned} {{\,\mathrm{spin}\,}}({\mathfrak {p}},\sigma ){{\,\mathrm{spin}\,}}({\mathfrak {p}},\sigma ^{-1}) = \prod _{v|2} (\alpha ,\alpha ^\sigma )_v, \end{aligned}$$

where $\alpha$ is a totally positive generator of ${\mathfrak {p}}^h$ and the product is taken over places *v* dividing 2.

#### Proof

This is essentially Lemma 11.1 in [5]. The proof uses the fact that

$$\begin{aligned} \prod _{v}(\alpha ,\alpha ^\sigma )_v=1. \end{aligned}$$

Since $\alpha \succ 0$, $(\alpha ,\alpha ^\sigma )_v=1$ for all infinite places *v*.

Consider *v*, a finite place not equal to ${\mathfrak {p}}$ or ${\mathfrak {p}}^\sigma$, and not dividing 2. Since $v\ne {\mathfrak {p}},{\mathfrak {p}}^\sigma$, we have $\alpha$ and $\alpha ^\sigma$ are non-zero modulo *v*. Consider the equation

$$\begin{aligned} \alpha ^\sigma x^2 \equiv 1 - \alpha y^2 \bmod v. \end{aligned}$$

The right hand side and the left hand side each take on $({\mathfrak {N}}(v)+1)/2$ values, so there is a solution by the pigeon hole principle. It can not be the case that both *x* and *y* are 0. Suppose $x\not \equiv 0 \bmod v$. Since *v* is prime to 2 and $x\not \equiv 0$, Hensel’s Lemma implies there exists a solution in the completion at *v*. Therefore $(\alpha , \alpha ^\sigma )_v=1$. If *y* is non-zero, a similar argument works.

Since $\alpha$ and $\alpha ^\sigma$ are relatively prime, $(\alpha ,\alpha ^\sigma )_{\mathfrak {p}}= \text {spin}({\mathfrak {p}},\sigma ^{-1})$ and $(\alpha ,\alpha ^\sigma )_{{\mathfrak {p}}^\sigma } = \text {spin}({\mathfrak {p}},\sigma )$. Then since $\prod _{v}(\alpha ,\alpha ^\sigma )_v=1$, we are done. $\square$

In this paper, we study the joint distribution of multiple spins ${{\,\mathrm{spin}\,}}({\mathfrak {p}}, \sigma )$, $\sigma \in S$, in a setting where there are in fact many $\sigma \in S$ such that $\sigma ^{-1}\in S$ as well. From the discussion above, we see that this might involve combining the work of Koymans and Milovic with the study of the products ${{\,\mathrm{spin}\,}}({\mathfrak {p}},\sigma ){{\,\mathrm{spin}\,}}({\mathfrak {p}},\sigma ^{-1})$ for various $\sigma$.

### Factorization and spin

The spin of prime ideals is related to the splitting behavior of *p* in both $K_+(p)$ and *K*(*p*) as we will see in Proposition 3 and Corollary 1.

Let $R_{\mathfrak {m}}^+$ denote the narrow ray class field over *K* of conductor ${\mathfrak {m}}$. Let ${\mathfrak {p}}$ be an odd prime of *K*. Recall from Definition 1 that Lemma 2 gives the existence of a unique quadratic subextension of $R_{{\mathfrak {p}}}^+/K$, denoted by $K({\mathfrak {p}})$. We have the following proposition for *K* satisfying properties (P1) and (C3).

#### Proposition 2

Assume the number field *K* satisfies properties (P1) and (C3). Let ${\mathfrak {p}}\subset {\mathcal {O}}$ be an odd prime ideal splitting completely in $K/{\mathbb {Q}}$. Let $\alpha \in {\mathcal {O}}$ be a totally positive generator of ${\mathfrak {p}}^h$. Then

$$\begin{aligned} K({\mathfrak {p}}) = K(\sqrt{u\alpha }) \end{aligned}$$

for some unit $u\in {\mathcal {O}}^\times$ well-defined modulo $({\mathcal {O}}^\times )^2$. We denote the unit class of *u* by ${\mathbf {u}}_K({\mathfrak {p}})\in {\mathcal {O}}^\times /({\mathcal {O}}^\times )^2$. Furthermore, ${\mathbf {u}}_K({\mathfrak {p}}^\sigma )={\mathbf {u}}_K({\mathfrak {p}})^\sigma$ for any $\sigma \in {{\,\mathrm{Gal}\,}}(K/{\mathbb {Q}})$.

#### Proof

The assumptions that *K* satisfies (P1) and (C3) ensure that we can define $K({\mathfrak {p}})$. Write $K({\mathfrak {p}}) = K(\sqrt{\beta })$ for $\beta \in {\mathcal {O}}$. The polynomial discriminant $d(1,\sqrt{\beta })$ satisfies

$$\begin{aligned} d(1,\sqrt{\beta }) = 4 \beta = {{\,\mathrm{disc}\,}}({\mathcal {O}}_L/{\mathcal {O}})({\mathcal {O}}_L: {\mathcal {O}}[\sqrt{\beta }])^2. \end{aligned}$$

where ${\mathcal {O}}_L$ denotes the ring of integers of $K(\sqrt{\beta })$.

If ${{\,\mathrm{ord}\,}}_{\mathfrak {q}}(4\beta )$ is odd for some prime ${\mathfrak {q}}$ of *K*, then ${{\,\mathrm{ord}\,}}_{\mathfrak {q}}({{\,\mathrm{disc}\,}}({\mathcal {O}}_L/{\mathcal {O}})({\mathcal {O}}_L: {\mathcal {O}}[\sqrt{\beta }])^2)$ is odd so ${{\,\mathrm{ord}\,}}_{\mathfrak {q}}({{\,\mathrm{disc}\,}}({\mathcal {O}}_L/{\mathcal {O}}))$ is odd. Then ${\mathfrak {q}}| {{\,\mathrm{disc}\,}}({\mathcal {O}}_L/{\mathcal {O}})$ so ${\mathfrak {q}}$ ramifies, and therefore ${\mathfrak {q}}={\mathfrak {p}}$. Since $h^+$ is odd and $K({\mathfrak {p}})/K$ is quadratic, ${\mathfrak {p}}$ ramifies in $K({\mathfrak {p}})$ so ${\mathfrak {p}}| {{\,\mathrm{disc}\,}}({\mathcal {O}}_L/{\mathcal {O}})$. Since $\beta$ is not a square in ${\mathcal {O}}$, ${{\,\mathrm{ord}\,}}_{\mathfrak {p}}({{\,\mathrm{disc}\,}}({\mathcal {O}}_L/{\mathcal {O}}))$ is odd. Therefore $(\beta ) = {\mathfrak {p}}{\mathfrak {a}}^2$ for some ideal ${\mathfrak {a}}$ of ${\mathcal {O}}$.

Raising to the power of the class number, $(\beta )^h = {\mathfrak {p}}^h {\mathfrak {a}}^{2h}$. Then since ${\mathfrak {a}}^{h}$ is principal, writing ${\mathfrak {a}}^{h} = (\delta )$, we have $(\beta )^h = (\alpha )(\delta )^2$. Then $\beta ^h = u\alpha \delta ^2$ for some unit $u \in {\mathcal {O}}^\times$. Since *h* is odd, $K(\sqrt{\beta }) = K(\sqrt{\beta ^h}) = K(\sqrt{u\alpha })$.

If $K({\mathfrak {p}})=K(\sqrt{v\alpha })= K(\sqrt{u\alpha })$ for $u,v\in {\mathcal {O}}^\times$, the Kummer pairing associates to this field the subgroup of $K^\times /(K^\times )^2$ given by $(K^\times \cap (L^\times )^2)/(K^\times )^2$, a cyclic subgroup of order 2. Both $u\alpha$ and $v\alpha$ generate this group so they are congruent modulo $(K^\times )^2$. Therefore *u* and *v* are equivalent in ${\mathcal {O}}^\times / ({\mathcal {O}}^\times )^2$. $\square$

#### Lemma 4

Suppose *K* is a number field and *h* is an odd number. Suppose ${\mathfrak {a}}$ and ${\mathfrak {b}}$ are distinct odd primes of *K*, and suppose $\alpha$ and $\beta$ are totally positive generators of ${\mathfrak {a}}^h$ and ${\mathfrak {b}}^h$, respectively, such that any prime above 2 is unramified in $K(\sqrt{\beta })/K$. Then

$$\begin{aligned} \left( \frac{\alpha }{{\mathfrak {b}}} \right) =\left( \frac{\beta }{{\mathfrak {a}}} \right) , \end{aligned}$$

where $(\cdot /\cdot )$ denotes the quadratic residue symbol in *K*.

#### Proof

Since *h* is odd and ${\mathfrak {b}}$ and ${\mathfrak {a}}$ are coprime, we have

2 $$\begin{aligned} \left( \frac{\alpha }{{\mathfrak {b}}} \right) = \left( \frac{\alpha }{{\mathfrak {b}}} \right) ^h = \left( \frac{\alpha }{{\mathfrak {b}}^h} \right) = \left( \frac{\alpha }{\beta } \right) . \end{aligned}$$

Similarly,

3 $$\begin{aligned} \left( \frac{\beta }{{\mathfrak {a}}} \right) = \left( \frac{\beta }{{\mathfrak {a}}} \right) ^h = \left( \frac{\beta }{{\mathfrak {a}}^h} \right) = \left( \frac{\beta }{\alpha } \right) . \end{aligned}$$

By the law of quadratic reciprocity for *K* [12, Theorem VI.8.3, p. 415], we have

$$\begin{aligned} \left( \frac{\alpha }{\beta } \right) =\left( \frac{\beta }{\alpha } \right) \prod _{v|2\infty }(\alpha ,\beta )_v, \end{aligned}$$

where $(\cdot , \cdot )_v$ is the Hilbert symbol on *K* and the product above is over all places *v* lying above 2 and infinity.

For each infinite place *v*, we have $(\alpha ,\beta )_v = 1$ since $\alpha$ is totally positive (and thus also positive in the embedding of *K* into ${\mathbb {R}}$ corresponding to *v*). For any place *v* lying above 2, we have $(\alpha ,\beta )_v = 1$ since $\alpha$ is coprime to 2 and any even prime is unramified in $K(\sqrt{\beta })/K$ (see [3, Exercise 2.8, p.352]). We thus deduce that

$$\begin{aligned} \left( \frac{\beta }{\alpha } \right) = \left( \frac{\alpha }{\beta } \right) , \end{aligned}$$

which in combination with (3) and (2) yields the desired result. $\square$

Given a rational prime *p*, fix a prime ${\mathfrak {p}}$ above *p* and a totally positive generator $\alpha$ of ${\mathfrak {p}}^h$. Recall from Definition 2 that $K_+(p)$ is the composite of $K_+({\mathfrak {p}}^{\sigma })$ as $\sigma$ varies over all elements of ${{\,\mathrm{Gal}\,}}(K/{\mathbb {Q}})$, where $K_+({\mathfrak {p}}^{\sigma }){:}{=} K(\sqrt{\alpha ^\sigma })$. As before, denote by $K({\mathfrak {p}}^{\sigma })$ the unique quadratic subextension of the narrow ray class field over *K* of conductor ${\mathfrak {p}}^{\sigma }$.

The factorization of *p* in $K_+(p)$ or *K*(*p*) is determined by the factorizations of ${\mathfrak {p}}$ in each $K_+({\mathfrak {p}}^{\sigma })$ or $K({\mathfrak {p}}^{\sigma })$ respectively, which is in turn determined by the spin of ${\mathfrak {p}}$ with respect to $\sigma$ or $\sigma ^{-1}$, respectively.

#### Proposition 3

Assume *K* satisfies properties (P1) and (C3). For a fixed odd prime ${\mathfrak {p}}$ of *K* that splits completely in $K/{\mathbb {Q}}$ and $\sigma$ non-trivial in ${{\,\mathrm{Gal}\,}}(K/{\mathbb {Q}})$, the following are equivalent.

1. $${{\,\mathrm{spin}\,}}({\mathfrak {p}},\sigma ) = 1 $$,
2. $$f_{K({\mathfrak {p}}^{\sigma })/K}({\mathfrak {p}})=1$$,
3. $$f_{K_+({\mathfrak {p}}^{\sigma ^{-1}})/K}({\mathfrak {p}})=1$$.

#### Proof

Let ${\mathfrak {q}}\ne {\mathfrak {p}}$ be an odd prime of *K* with $\beta \in {\mathcal {O}}$ a generator of ${\mathfrak {q}}^h$ where *h* is the class number of *K*. Let $L = K(\sqrt{\beta })$. We will prove that $(\beta /{\mathfrak {p}}) =1$ if and only if $f_{L|K}({\mathfrak {p}}) = 1$. The result will then be established by choosing suitable $\beta$ and ${\mathfrak {q}}$.

If $m>0$ is the minimal positive integer such that ${\mathfrak {q}}^m$ is principal and if ${\mathfrak {q}}^r$ is principal for any $r>0$, then we can write $r = a + ml$ for $l>0$ and $0 \le a < m$. Then ${\mathfrak {q}}^r = {\mathfrak {q}}^a {\mathfrak {q}}^{ml}$ and since ${\mathfrak {q}}^r$ and ${\mathfrak {q}}^{m}$ are principal, so is ${\mathfrak {q}}^a$. Since *m* is the minimal such positive integer, $a=0$ so *m*|*r*. That is, any power of ${\mathfrak {q}}$ that is principal must be a power of ${\mathfrak {q}}^m$. In particular since *h* is odd, *m* is odd and if ${\mathfrak {q}}^{2l}$ is principal, then ${\mathfrak {q}}^l$ is principal.

Write ${\mathfrak {q}}^h = {\mathfrak {q}}^m{\mathfrak {q}}^{2l}$ where *m* is the minimal positive integer such that ${\mathfrak {q}}^m$ is principal. By Lemma 1, we can write $(\gamma ) = {\mathfrak {q}}^m$ for $\gamma \in {\mathcal {O}}$ with the same signature as $\beta$. Then ${\mathfrak {q}}^h = {\mathfrak {q}}^m{\mathfrak {q}}^{2l}$ becomes $(\beta ) = (\gamma ) (\delta )^2$ for $\delta$ a generator of ${\mathfrak {p}}^l$. Since the signatures of $\beta$ and $\gamma$ match, $\beta = \gamma \delta ^2$. Therefore $L = K(\sqrt{\beta }) = K(\sqrt{\gamma })$.

Here ${\mathcal {O}}$ denotes the ring of integers of *K* and ${\mathcal {O}}_L$ denotes the ring of integers of *L*. The polynomial discriminant $d(1, \sqrt{\gamma }) = 4 \gamma$ so

$$\begin{aligned} 4 \gamma = {{\,\mathrm{disc}\,}}({\mathcal {O}}_L/ {\mathcal {O}}) ({\mathcal {O}}_L: {\mathcal {O}}[\sqrt{\gamma }])^2. \end{aligned}$$

If ${\mathfrak {q}}| ({\mathcal {O}}_L: {\mathcal {O}}[\sqrt{\gamma }])$ then since $({\mathcal {O}}_L: {\mathcal {O}}[\sqrt{\gamma }])$ is an integer, $t {:}{=} {{\,\mathrm{ord}\,}}_{\mathfrak {q}}({\mathcal {O}}_L: {\mathcal {O}}[\sqrt{\gamma }])$ must be such that ${\mathfrak {q}}^t$ is principal. Then *m*|*t* but since ${\mathfrak {q}}$ is odd,

$$\begin{aligned} {{\,\mathrm{ord}\,}}_{\mathfrak {q}}({{\,\mathrm{disc}\,}}({\mathcal {O}}_L/ {\mathcal {O}}) ({\mathcal {O}}_L: {\mathcal {O}}[\sqrt{\gamma }])^2) \ge 2t > m = {{\,\mathrm{ord}\,}}_{\mathfrak {q}}(4 \gamma ). \end{aligned}$$

This is a contradiction, so ${\mathfrak {q}}\not \mid ({\mathcal {O}}_L: {\mathcal {O}}[\sqrt{\gamma }])$. Therefore $({\mathcal {O}}_L: {\mathcal {O}}[\sqrt{\gamma }]) | 2$. Then since $p ={\mathfrak {N}}({\mathfrak {p}})$ is odd and ${{\mathcal {O}}[\sqrt{\gamma }]}/{{\mathfrak {p}}}$ and ${{\mathcal {O}}_L}/{{\mathfrak {p}}}$ are both vector spaces over ${\mathbb {Z}}/p$, these two rings are isomorphic. As the quotient of $({\mathcal {O}}/{\mathfrak {p}})[x]$ by the polynomial $x^2 - \gamma$ considered in $({\mathcal {O}}/{\mathfrak {p}})[x]$ is isomorphic to ${{\mathcal {O}}[\sqrt{\gamma }]}/{{\mathfrak {p}}}$,

4 $$\begin{aligned} ({\mathcal {O}}/{\mathfrak {p}})[x] / (x^2 - \gamma ) \cong {{\mathcal {O}}_L}/{{\mathfrak {p}}}. \end{aligned}$$

The quadratic residue $(\gamma / {\mathfrak {p}})$ is equal to 1 exactly when the polynomial $x^2 - \gamma$ factors in $({\mathcal {O}}/{\mathfrak {p}})[x]$. Since ${\mathfrak {p}}\not \mid (\gamma )$, the polynomial $x^2 - \gamma$ cannot factor into the square of an irreducible polynomial. The irreducible factors of $x^2 - \gamma$ in $({\mathcal {O}}/{\mathfrak {p}})[x]$ correspond bijectively to the maximal ideals of $({\mathcal {O}}/{\mathfrak {p}})[x] / (x^2 - \gamma )$. Then since ${\mathfrak {p}}\not \mid (\gamma )$, we may deduce that $(\gamma / {\mathfrak {p}}) = 1$ if and only if there are exactly two maximal ideals of $({\mathcal {O}}/{\mathfrak {p}})[x] / (x^2 - \gamma )$. Applying the isomorphism in line (4), this is true if and only if ${\mathcal {O}}_L/{\mathfrak {p}}$ has exactly two maximal ideals. Maximal ideals of ${\mathcal {O}}_L/{\mathfrak {p}}$ correspond bijectively to the irreducible factors of ${\mathfrak {p}}$ in ${\mathcal {O}}_L$. Therefore $(\gamma / {\mathfrak {p}}) = 1$ if and only if $f_{L|K}({\mathfrak {p}}) =1$. Since $\beta = \gamma \delta ^2$, $(\beta / {\mathfrak {p}}) = (\gamma / {\mathfrak {p}})$. This concludes the first part of the proof, showing that $(\beta /{\mathfrak {p}}) =1$ if and only if $f_{L|K}({\mathfrak {p}}) = 1$.

Setting $\beta = (u\alpha )^\sigma$ for *u* in the unit class ${\mathbf {u}}_K({\mathfrak {p}})$ and ${\mathfrak {q}}= {\mathfrak {p}}^\sigma$, by Proposition 2, $L = K(\sqrt{\beta }) = K(\sqrt{(u\alpha )^\sigma }) = K({\mathfrak {p}}^\sigma )$. Since $K(\sqrt{(u\alpha )^\sigma })$ is contained in $R_{{\mathfrak {p}}^\sigma }^+$, no prime above 2 is ramified in the extension $K(\sqrt{(u\alpha )^\sigma })/K$, so applying Lemma 4,

$$\begin{aligned}(\beta /{\mathfrak {p}}) = ((u\alpha )^\sigma / {\mathfrak {p}}) = (\alpha / {\mathfrak {p}}^\sigma ) = {{\,\mathrm{spin}\,}}({\mathfrak {p}},\sigma ),\end{aligned}$$

proving (2.) is equivalent to part (1.).

Alternatively, if we set $\beta = \alpha ^{\sigma ^{-1}}$ and ${\mathfrak {q}}= {\mathfrak {p}}^{\sigma ^{-1}}$, then $L = K(\sqrt{\beta }) = K({\mathfrak {p}}^{\sigma ^{-1}})$ and since $(\alpha ^{\sigma ^{-1}}/{\mathfrak {p}}) = (\alpha / {\mathfrak {p}}^\sigma ) = {{\,\mathrm{spin}\,}}({\mathfrak {p}},\sigma )$, this proves that part (3.) is equivalent to part (1.). $\square$

#### Corollary 1

For a fixed odd rational prime *p* splitting completely in $K/{\mathbb {Q}}$, the residue field degrees of *p* in the extensions $K(p)/{\mathbb {Q}}$ and $K_+(p)/{\mathbb {Q}}$ are equal to 1 if and only if ${{\,\mathrm{spin}\,}}({\mathfrak {p}},\sigma )=1$ for all non-trivial $\sigma \in {{\,\mathrm{Gal}\,}}(K/{\mathbb {Q}})$ for ${\mathfrak {p}}$ a prime of *K* above *p*. Otherwise these residue field degrees are equal to 2.

#### Proof

$f_{K(p)/{\mathbb {Q}}}(p)=1$ exactly when $f_{K({\mathfrak {p}}^{\sigma })/K}({\mathfrak {p}})=1$ for all $\sigma \in {{\,\mathrm{Gal}\,}}(K/{\mathbb {Q}})$. Similarly, $f_{K_+(p)/{\mathbb {Q}}}(p)=1$ exactly when $f_{K_+({\mathfrak {p}}^{\sigma ^{-1}})/K}({\mathfrak {p}})=1$ for all $\sigma \in {{\,\mathrm{Gal}\,}}(K/{\mathbb {Q}})$. Apply Proposition 3. If the residue field degrees are not equal to 1 then they are equal to 2 by Lemma 3. $\square$

## A consequence of the Chebotarev Density theorem

In this section, we use the Chebotarev Density Theorem to prove that the primes of *K* are equidistributed in ${\mathbf {M}}_4$ as defined below, where the mapping takes primes to a totally positive generator considered in ${\mathbf {M}}_4$. This contributes toward the density *d*(*R*|*S*) of rational primes *p* that satisfy the spin relation,

$$\begin{aligned} {{\,\mathrm{spin}\,}}({\mathfrak {p}},\sigma ) {{\,\mathrm{spin}\,}}({\mathfrak {p}},\sigma ^{-1})=1 \quad \text {for all non-trivial } \sigma \in {{\,\mathrm{Gal}\,}}(K/{\mathbb {Q}}), \end{aligned}$$

where ${\mathfrak {p}}$ is a prime of *K* above *p*, restricted to the rational primes splitting completely in $K/{\mathbb {Q}}$. We will also give this density restricted modulo $4{\mathbb {Z}}$. Theorem 4 and Proposition 5 together give the density of such primes satisfying the spin relation.

### Definition 4

For *q* a power of 2, define

$$\begin{aligned} {\mathbf {M}}_q:= ({\mathcal {O}}/q{\mathcal {O}})^\times / \left( ({\mathcal {O}}/q{\mathcal {O}})^\times \right) ^2. \end{aligned}$$

Note that ${\mathbf {M}}_q$ is a group with a natural action from ${{\,\mathrm{Gal}\,}}(K/{\mathbb {Q}})$.

### Proposition 4

Let *K* be a number field satisifying (C1) and (C4). Then

1. ${\mathbf {M}}_4\cong ({\mathbb {Z}}/2)^n$ as a ${\mathbb {Z}}/2$-vector space,
2. the invariants of the action of ${{\,\mathrm{Gal}\,}}(K/{\mathbb {Q}})$ on ${\mathbf {M}}_4$ are exactly $\pm 1$.

### Proof

Let $$U_m{:}{=} ({\mathcal {O}}/m)^\times $$.

1. Fix a set of representatives ${\mathcal {R}}$ for ${\mathcal {O}}/2$ in ${\mathcal {O}}$. Let ${\mathcal {R}}^\times$ be a subset of ${\mathcal {R}}$ containing representatives for $({\mathcal {O}}/2)^\times$. Observe that $\{ x+2y: x \in {\mathcal {R}}^\times , y \in {\mathcal {R}} \}$ is a set of representatives for $U_4$ and $\#U_4 = 2^n(2^n-1)$. Therefore elements of $U_4^2$ are of the form $(x+2y)^2 \equiv x^2 \bmod 4{\mathcal {O}}$ for $x \in {\mathcal {R}}^\times$ and $y \in {\mathcal {R}}$. Since $\#({\mathcal {O}}/2)^\times = 2^n -1$ is odd, the squaring map on $U_2=({\mathcal {O}}/2)^\times$ is surjective and so $\#U_4^2 = 2^n-1$. Therefore $\#{\mathbf {M}}_4= \#U_4/\#U_4^2 = 2^n$. Since ${\mathbf {M}}_4$ is formed by taking the quotient of $U_4$ modulo squares, ${\mathbf {M}}_4$ is a direct product of cyclic groups of order 2.
  For any $\alpha \in {\mathcal {O}}$ coprime to 2, write $[\alpha ]$ as the projection of $\alpha {\mathcal {O}}$ in ${\mathbf {M}}_4$. Since every $x\in {\mathcal {R}}^\times$ is a square in $U_2$, we can write down the isomorphism explicitly as
  5 $$\begin{aligned} {\mathbf {M}}_4\rightarrow {\mathcal {O}}/2\cong {\mathbb {F}}_{2^n}\qquad [x+2y]=[1+2x^{-1}y]\mapsto x^{-1}y. \end{aligned}$$
  We see that ${\mathbf {M}}_4=\{[1+2y]:y\in {\mathcal {O}}/2\}$.
2. Let $\sigma$ be a generator of ${{\,\mathrm{Gal}\,}}(K/{\mathbb {Q}})$. The action of $\sigma$ on $[1+2y]\in {\mathbf {M}}_4$, simply maps *y* to $y^{\sigma }$. Then we see that $y\equiv y^\sigma \bmod {\mathcal {O}}/2$ if and only if $y\equiv 0$ or $1\bmod {\mathcal {O}}/2$. These correspond to $\pm 1$ in ${\mathbf {M}}_4$.

$$\square $$

### Lemma 5

Let *K* be a number field such that 2 is inert in $K/{\mathbb {Q}}$. The Hilbert symbol $(\text{[ }1.25ex]\mathbf{\cdot }, \text{[ }1.25ex]\mathbf{\cdot })_2$ is well-defined on ${\mathbf {M}}_4$.

### Proof

We show that $(\alpha ,\beta )_2=(\alpha +4B,\beta )_2$ for any $B\in {\mathcal {O}}$ coprime to 2, which implies that $(\text{[ }1.25ex]\mathbf{\cdot }, \text{[ }1.25ex]\mathbf{\cdot })_2$ is well-defined on $({\mathcal {O}}/4{\mathcal {O}})^{\times }\times ({\mathcal {O}}/4{\mathcal {O}})^{\times }$. Suppose $B\in {\mathcal {O}}$ is coprime to 2. It suffices to show that $(\alpha ,\beta )_2=1$ implies $(\alpha +4B,\beta )_2=1$. Assuming $(\alpha ,\beta )_2=1$, we can take $x,y,z\in {\mathcal {O}}$ not all divisible by 2 satisfying $x^2-\alpha y^2=\beta z^2\bmod 8$. Since all elements of $({\mathcal {O}}/2)^\times$ are squares in $({\mathcal {O}}/2)^\times$, there exists $C,D\in {\mathcal {O}}$ such that $C^2\equiv \alpha ^{-1}\beta B\bmod 2$ and $D^2\equiv \alpha ^{-1}\beta ^{-1}B\bmod 2$. Take $X=x+2Cz$, $y=Y$ and $Z=z+2Dx$, then one can check that $X^2-(\alpha +4B) Y^2\equiv \beta Z^2\bmod 8$. Therefore $(\alpha +4B,\beta )_2=1$ by Hensel’s lifting the solution (*X*, *Y*, *Z*). $\square$

### Lemma 6

Let *K* be a number field such that 2 is inert in $K/{\mathbb {Q}}$. The Hilbert symbol $(\text{[ }1.25ex]\mathbf{\cdot },\text{[ }1.25ex]\mathbf{\cdot })_2$ is non-degenerate on ${\mathbf {M}}_4$.

### Proof

Fix some $\alpha \in {\mathcal {O}}$ coprime to 2. We claim that $(\alpha +4B,2)_2=1$ for some $B\in {\mathcal {O}}$. Since $({\mathcal {O}}/2)^\times$ contains all its squareroots, there exist some $\gamma , z\in {\mathcal {O}}$ such that $\alpha \equiv \gamma ^2-2z^2\bmod 4$. Write $x=\gamma +2x'$ for some $x'\in {\mathcal {O}}$, set $B=x'\gamma +x'^2$ and $y=1$. Then $x^2-(\alpha +4B)y^2\equiv 2z^2\bmod 8$. This proves our claim.

Now suppose $(\alpha ,\beta )_2=1$ for all $\beta \in {\mathcal {O}}$ coprime to 2. Then taking *B* from the above claim, $(\alpha +4B,\beta )_2=1$ holds for all $\beta \in {\mathcal {O}}$ coprime to 2 by Lemma 5, and for all $\beta \in {\mathcal {O}}$ divisible by 2, by the above claim. Since the Hilbert symbol is non-degenerate on $K_2^\times /(K_2^\times )^2$ [13, Chapter XIV, Proposition 7], this implies that $\alpha +4B\in {\mathcal {O}}^2$. Hence $[\alpha ]=[\alpha +4B]$ is trivial in ${\mathbf {M}}_4$. $\square$

For ${\mathfrak {m}}$ an ideal of *K*, let ${\mathscr {P}}_K^{\mathfrak {m}}$ denote the set of prime ideals of ${\mathcal {O}}$ co-prime to ${\mathfrak {m}}$. For *K* a totally real number field satisfying (C3), we can define the following map.

### Definition 5

For *q* a power of 2, define the map

$$\begin{aligned} {\mathbf {r}}_q:&{\mathscr {P}}_K^{2}\rightarrow {\mathbf {M}}_q \\&{\mathfrak {p}}\mapsto \alpha \end{aligned}$$

where $\alpha \in {\mathcal {O}}$ is a totally positive generator of the principal ideal ${\mathfrak {p}}^{h}$.

By Lemma 1 since *K* is totally real with odd narrow class number, all principal ideals have a totally positive generator and ${\mathcal {O}}^{\times }_{+} = \left( {\mathcal {O}}^{\times }\right) ^2$. Since squares are trivial in ${\mathbf {M}}_4$ by definition the map ${\mathbf {r}}_q$ is well-defined. We also note that ${\mathbf {r}}_q$ commutes with the Galois action, i.e. ${\mathbf {r}}_q({\mathfrak {p}}^\sigma ) = {\mathbf {r}}_q({\mathfrak {p}})^\sigma$ for all $\sigma \in {{\,\mathrm{Gal}\,}}(K/{\mathbb {Q}})$.

For ${\mathfrak {m}}$ an ideal of ${\mathcal {O}}$, let $J_K^{\mathfrak {m}}$ denote the group of fractional ideals of *K* prime to ${\mathfrak {m}}$.

### Lemma 7

[ [9, Lemma 3.5]] For *K* totally real with $h^+$ odd, the homomorphism $J_K^2 \rightarrow {\mathbf {M}}_q$ induced by ${\mathbf {r}}_q$ induces a surjective homomorphism,

$$\begin{aligned} \varphi _q: \mathrm {Cl}^+_{q} \rightarrow {\mathbf {M}}_q. \end{aligned}$$

### Proof

This result is proven in [9, 3.5] and while stated with more assumptions there, the same proof holds with only the assumptions stated here. For clarity, we expand upon the proof of surjectivity. Let

$$\begin{aligned}&K_{\mathfrak {m}}:=\{a\in K^\times : {{\,\mathrm{ord}\,}}_2(a)=0\}, \quad \text {and}\\&K_{{\mathfrak {m}},1}:=\{a \in K^\times : {{\,\mathrm{ord}\,}}_2(a-1)\ge {{\,\mathrm{ord}\,}}_2(q), {a}\succ 0\} \end{aligned}$$

We have the following commutative diagram of homomorphisms. The homomorphism $\psi _0$ and the isomorphism *i* are induced by the exact sequence and canonical isomorphism from class field theory as given in [10, V.1.7].
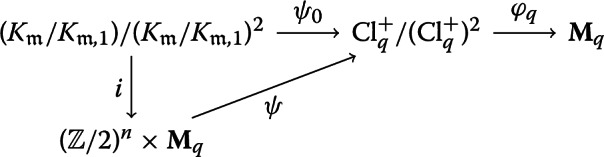


Fix $X\in {\mathbf {M}}_q$. Consider $(0,X)\in ({\mathbb {Z}}/2)^n\times {\mathbf {M}}_q$. Since *i* is an isomorphism, there exists an element in $(K_{\mathfrak {m}}/K_{{\mathfrak {m}},1})/(K_{\mathfrak {m}}/K_{{\mathfrak {m}},1})^2$ represented by $\beta \in K^\times$ such that $i(\beta )=(0,X)$. Since $i(\beta )$ maps to 0 in the projection to $({\mathbb {Z}}/2)^n$, $\beta$ is totally positive. Since ${\beta }\succ 0$, we can choose $a,b\in {\mathcal {O}}$ totally positive such that $\beta =a/b$. (Writing $\beta =a/b$ for any $a,b\in {\mathcal {O}}$, one could then consider $\beta =a^2/ab$). Then $X=[ab^{-1}]$ by the canonical isomorphism in [10, V.1.7].

The map $\psi _0$ takes $\beta$ to the class in $\mathrm {Cl}_{q}^+/(\mathrm {Cl}_{q}^+)^2$ represented by the fractional ideal $(a)(b)^{-1}$. Factoring the ideal (*c*) for any totally positive element $c \in {\mathcal {O}}$ and applying the homomorphism $\varphi _q$ gives $[c^h] \in {\mathbf {M}}_q$ which is equivalent to $[c] \in {\mathbf {M}}_q$ since *h* is odd. Then since *a* and *b* are totally positive, $\varphi _q((a)(b)^{-1})=[ab^{-1}]=X$ and so $\varphi _q$ is surjective. $\square$

Let $S'$ denote the set of odd primes ${\mathfrak {p}}$ of *K* with inertia degree $f_{K/{\mathbb {Q}}}({\mathfrak {p}})=1$.

### Lemma 8

[[9, Lemma 4.3]] Assume *K* satisfies (P1), (C3), and (C4).

1. For any $\alpha \in {\mathbf {M}}_4$, the density of primes ${\mathfrak {p}}$ of *K* such that $\varphi _4({\mathfrak {p}})=\alpha$ is $\frac{1}{2^n}$. That is,
  $$\begin{aligned} d({\mathbf {r}}_4^{-1}(\alpha )) = \frac{1}{\#{\mathbf {M}}_4} = \frac{1}{2^n}. \end{aligned}$$
2. Furthermore, the density does not change when we restrict to primes of *K* that split completely in $K/{\mathbb {Q}}$. That is,
  $$\begin{aligned} d({\mathbf {r}}_4^{-1}(\alpha ) \cap S' | S') = \frac{1}{\#{\mathbf {M}}_4} = \frac{1}{2^n}. \end{aligned}$$

### Proof

See [9, Lemma 4.3]. There the result is stated with more assumptions, but the same proof holds more generally. $\square$

### Definition 6

Assume *K* satisfies (P1), (C3). Let $\alpha \in {\mathbf {M}}_4$. Let ${\mathfrak {p}}$ be an odd prime of *K* such that ${\mathbf {r}}_4({\mathfrak {p}})=\alpha$. The map

$$\begin{aligned} {\mathbf {N}}: {\mathbf {M}}_4&\rightarrow ({\mathbb {Z}}/4)^\times \\ \alpha&\mapsto {\mathfrak {N}}_{K/{\mathbb {Q}}}({\mathfrak {p}}) \bmod 4{\mathbb {Z}}\end{aligned}$$

is well-defined and ${\mathbf {N}}(\alpha )={\mathbf {N}}(\alpha ^\sigma )$ for all $\sigma \in {{\,\mathrm{Gal}\,}}(K/{\mathbb {Q}})$.

### Proof

By Lemma 7, the map ${\mathbf {r}}_4: {\mathscr {P}}_K^{2} \rightarrow {\mathbf {M}}_4$ from Definition 5 induces a surjective group homomorphism

$$\begin{aligned} \varphi _4: \mathrm {Cl}_4^+ \twoheadrightarrow {\mathbf {M}}_4. \end{aligned}$$

Define $H{:}{=} {{\,\mathrm{Art}\,}}(\ker (\varphi _4))$ where ${{\,\mathrm{Art}\,}}$ denotes the Artin map from $\mathrm {Cl}_4^+$ to ${{\,\mathrm{Gal}\,}}(R_4^+/K)$ with $R_4^+$ denoting the narrow ray class field over *K* of conductor 4.

Define *L* to be the fixed field of *H* in ${{\,\mathrm{Gal}\,}}(R_4^+/K)$. Then $\varphi _4$ induces a canonical isomorphism

$$\begin{aligned} {{\,\mathrm{Gal}\,}}(L/K)\cong {\mathbf {M}}_4. \end{aligned}$$

Then for any $\alpha \in {\mathbf {M}}_4$, by applying the Chebotarev Density Theorem to the element of ${{\,\mathrm{Gal}\,}}(L/K)$ corresponding to $\alpha$ via this isomorphism, there exists a prime ${\mathfrak {p}}\in {\mathscr {P}}_K^{2}$ with $\varphi _4({\mathfrak {p}}) = \alpha$.

Let ${\mathfrak {p}}$ and ${\mathfrak {q}}$ be odd primes of *K* such that ${\mathbf {r}}_4({\mathfrak {p}})={\mathbf {r}}_4({\mathfrak {q}})$. Let $\alpha$ be a totally positive generator of ${\mathfrak {p}}^h$ and let $\beta$ be a totally positive generator of ${\mathfrak {q}}^h$, where *h* is the odd class number of *K*, which is odd by assumption. Since ${\mathbf {r}}_4({\mathfrak {p}})={\mathbf {r}}_4({\mathfrak {q}})$, $\alpha \equiv \beta$ in ${\mathbf {M}}_4$. Then $\alpha \equiv \beta \gamma ^2\bmod 4{\mathcal {O}}$ for some $\gamma \in {\mathcal {O}}$. Since $\alpha ^\sigma \equiv \beta ^\sigma (\gamma ^\sigma )^2\bmod 4{\mathcal {O}}$ for all $\sigma \in {{\,\mathrm{Gal}\,}}(K/{\mathbb {Q}})$, taking norms ${\mathfrak {N}}(\alpha ) \equiv {\mathfrak {N}}(\beta ) {\mathfrak {N}}(\gamma )^2 \bmod 4{\mathcal {O}}$. Since the norms are in ${\mathbb {Z}}$, ${\mathfrak {N}}(\alpha ) \equiv {\mathfrak {N}}(\beta )\bmod 4{\mathbb {Z}}$. $\square$

We now state an extended version of Lemma 8 that handles the densities restricted to primes of a fixed congruence class modulo $4{\mathbb {Z}}$.

For a fixed sign $\mu \in \{\pm \}$, let $S_{\mu }'$ denote the set of primes ${\mathfrak {p}}\in S'$ with ${\mathfrak {N}}{({\mathfrak {p}})}\equiv \mu 1 \bmod 4{\mathbb {Z}}$. In other words $S_{\mu }'$ is the set of primes of *K* laying above rational primes in $S_\mu$.

### Lemma 9

Assume *K* satisfies conditions (C1)-(C4). For any $\alpha \in {\mathbf {M}}_4$ and for a fixed sign $\mu \in \{\pm \}$, the density of ${\mathfrak {p}}\in S_{\mu }'$ such that $\varphi _4({\mathfrak {p}})=\alpha$ is given by

$$\begin{aligned} d({\mathbf {r}}_4^{-1}(\alpha ) \cap S_{\mu }'|S_{\mu }') = \left\{ \begin{array}{ll} \frac{1}{2^{n-1}} &{} \text {if } {\mathbf {N}}(\alpha ) = \mu 1 \bmod 4\\ 0 &{} \text {otherwise.} \end{array} \right. \end{aligned}$$

### Proof

Let $K_{4,1}{:}{=}\{\alpha \in K^\times : {{\,\mathrm{ord}\,}}_2(\alpha -1)\ge 2, \alpha \succ 0\}$ and ${\mathcal {O}}^\times _{4,1} {:}{=} K_{4,1} \cap {\mathcal {O}}^\times$. Since ${\mathcal {O}}^\times _+=({\mathcal {O}}^\times )^2$,

$$\begin{aligned} ({\mathcal {O}}^\times :{\mathcal {O}}^\times _{4,1}) = ({\mathcal {O}}^\times :{\mathcal {O}}^\times _+)({\mathcal {O}}^\times _+:{\mathcal {O}}^\times _{4,1}) = 2^n(({\mathcal {O}}^\times )^2:{\mathcal {O}}^\times _{4,1}). \end{aligned}$$

Therefore by [10, V.1.7], the order of ${{\,\mathrm{Gal}\,}}(R_4^+/K)$ divides $h2^n(2^n-1)$.

As in the proof following Definition 6 and with *L* as defined there, recall that ${\mathbf {r}}_4$ induces a canonical isomorphism ${{\,\mathrm{Gal}\,}}(L/K) \cong {\mathbf {M}}_4.$ Then $[L:K]=2^n$ by Proposition 4. Therefore $[R_4^+:L]$ is odd. Let *F* denote the composite of *K* and ${\mathbb {Q}}(\zeta _4)$. Since $[K:{\mathbb {Q}}]$ is odd, $[F:K]=2$. Since $K \subseteq F \subseteq R_4^+$ and $[R_4^+:L]$ is odd, $F\subseteq L$ and $[L:F]=2^{n-1}$.

For *T*/*E* a Galois extension of conductor dividing ${\mathfrak {m}}$, let *p* be a prime of *E*, and let $\tau \in {{\,\mathrm{Gal}\,}}(T/E)$. Let (*p*, *T*/*E*) denote the conjugacy class of ${{\,\mathrm{Gal}\,}}(T/E)$ containing the Frobenius of ${\mathfrak {p}}$ where ${\mathfrak {p}}$ is a prime of *T* above *p*. Let

$$\begin{aligned} {\mathcal {A}}_{T|E}^{E}(\tau )&{:}{=} \{ p\in {\mathscr {P}}_E^{{\mathfrak {m}}}: (p,T/E) = \left<\tau \right>\},\\ {\mathcal {A}}_{T|E}^{T}(\tau )&{:}{=} \{ {\mathfrak {p}}\in {\mathscr {P}}_T^{{\mathfrak {m}}}: {\mathfrak {p}}\text { lies above }p\in {\mathcal {A}}_{T|E}^{E}(\tau ) \}. \end{aligned}$$

Fix $\mu \in \{\pm \}$. Let $\tau _-$ denote the nontrivial element of ${{\,\mathrm{Gal}\,}}(F/K)$ and let $\tau _+$ denote the trivial element of ${{\,\mathrm{Gal}\,}}(F/K)$ so that ${\mathfrak {N}}({\mathfrak {p}}) = \mu 1 \bmod 4$ exactly when ${\mathfrak {p}}\in {\mathcal {A}}_{F|K}^{K}(\tau _\mu )$. Furthermore $S_\mu ' = S' \cap {\mathcal {A}}_{F|K}^{K}(\tau _\mu )$.

Fix $\alpha \in {\mathbf {M}}_4$ and let $\sigma \in {{\,\mathrm{Gal}\,}}(L/K)$ corresponding to $\alpha$ via the isomorphism induced by ${\mathbf {r}}_4$ given in the proof following Definition 6. Then ${\mathbf {r}}_4^{-1}(\alpha ) = {\mathcal {A}}_{L|K}^{K} (\sigma )$.

Letting ${\overline{\sigma }}$ denote the image of $\sigma$ in the natural surjection to ${{\,\mathrm{Gal}\,}}(F/K)$,

$$\begin{aligned} {\mathcal {A}}_{L|K}^{K} (\sigma ) \cap {\mathcal {A}}_{F|K}^{K} (\tau _\mu ) = \left\{ \begin{array}{ll} {\mathcal {A}}_{L|K}^{K} (\sigma ) &{} \text { if } {\overline{\sigma }} = \tau _\mu , \\ 0 &{} \text {otherwise.} \\ \end{array} \right. \end{aligned}$$

By the Chebotarev Density Theorem, $d({\mathcal {A}}_{L|K}^{K} (\sigma ) ) = \frac{1}{2^n}$. Since $S'$ has density 1, restricting densities of primes in *K* to those that split completely in $K/{\mathbb {Q}}$ does not change the density. Therefore

$$\begin{aligned} d({\mathbf {r}}_4^{-1}(\alpha ) \cap S_\mu ' | S')&= d({\mathcal {A}}_{L|K}^{K} (\sigma ) \cap {\mathcal {A}}_{F|K}^{K} (\tau _\mu ) \cap S' | S' ) \\&= d({\mathcal {A}}_{L|K}^{K} (\sigma ) \cap {\mathcal {A}}_{F|K}^{K} (\tau _\mu )) \\&= \left\{ \begin{array}{ll} \frac{1}{2^n} &{} \text { if } {\overline{\sigma }} = \tau _\mu , \\ 0 &{} \text {otherwise} \\ \end{array} \right. \end{aligned}$$

and similarly $d(S_\mu '|S') = d( {\mathcal {A}}_{F|K}^{K}(\tau _\mu ) \cap S'| S') = d( {\mathcal {A}}_{F|K}^{K}(\tau _\mu ))$ which is equal to $\frac{1}{2}$ by the Chebotarev Density Theorem. Therefore

$$\begin{aligned} d({\mathbf {r}}_4^{-1}(\alpha ) \cap S_\mu ' | S_\mu ') = \frac{ d({\mathbf {r}}_4^{-1}(\alpha ) \cap S_\mu ' | S') }{ d(S_\mu ' | S')} = \left\{ \begin{array}{ll} \frac{1}{2^{n-1}} &{} \text { if } {\overline{\sigma }} = \tau _\mu , \\ 0 &{} \text {otherwise.} \\ \end{array} \right. \end{aligned}$$

For ${\mathfrak {p}}\in {\mathbf {r}}_4^{-1}(\alpha )$, the condition that ${\mathbf {N}}(\alpha ) = \mu 1 \bmod 4$ means that ${\mathfrak {N}}({\mathfrak {p}}) = \mu 1 \bmod 4$. This is equivalent to the condition that ${\mathfrak {p}}\in {\mathcal {A}}_{F|K}^{K}(\tau _\mu )$. Since ${\mathfrak {p}}\in {\mathbf {r}}_4^{-1}(\alpha ) = {\mathcal {A}}_{L|K}^{K}(\sigma )$, the condition that ${\mathfrak {p}}\in {\mathcal {A}}_{F|K}^{K}(\tau _\mu )$ is true exactly when ${\overline{\sigma }} = \tau _\mu$. This completes the proof. $\square$

Recall that Proposition 1 states that for ${\mathfrak {p}}$ a prime of *K* with totally positive generator $\alpha \in {\mathcal {O}}$, and for $\sigma \in {{\,\mathrm{Gal}\,}}(K/{\mathbb {Q}})$ such that ${\mathfrak {p}}$ and ${\mathfrak {p}}^\sigma$ are relatively prime,

$$\begin{aligned} {{\,\mathrm{spin}\,}}({\mathfrak {p}},\sigma ){{\,\mathrm{spin}\,}}({\mathfrak {p}},\sigma ^{-1}) = (\alpha ,\alpha ^\sigma )_2. \end{aligned}$$

This motivates the following definition.

### Definition 7

( [9, Theorem 5.1] ) Assume *K* is Galois with abelian Galois group and satisfies (C4). Let $\alpha \in {\mathcal {O}}$ denote a representative of $[\alpha ]\in$ ${\mathbf {M}}_{4}$. Define the map

$$\begin{aligned} {\star }: { {\mathbf {M}}_{4} }&\rightarrow \{\pm 1\} \\ [\alpha ]&\mapsto \left\{ \begin{array}{l l} 1 &{} \quad \text {if } (\alpha ,\alpha ^\sigma )_2 = 1 \text { for all non-trivial } \sigma \in {{\,\mathrm{Gal}\,}}(K/{\mathbb {Q}}), \\ -1 &{} \quad \text {otherwise.}\\ \end{array} \right. \end{aligned}$$

Observe that $\star$ is a well-defined map by Lemma 5. If (6) holds for some $\alpha \in {\mathcal {O}}$, then it holds for $\alpha ^{\sigma }$ for any $\sigma \in {{\,\mathrm{Gal}\,}}(K/{\mathbb {Q}})$. Therefore $\star (\alpha )=\star (\alpha ^\sigma )$ for all $\sigma \in {{\,\mathrm{Gal}\,}}(K/{\mathbb {Q}})$.

Recall the map ${\mathbf {N}}: {\mathbf {M}}_4 \rightarrow \pm 1$ from Definition 6. Let $\star _+$ denote the restriction of $\star$ to

$$\begin{aligned}{\mathbf {M}}_4^+{:}{=} \{\alpha \in {\mathbf {M}}_4: {\mathbf {N}}(\alpha )=1\}\end{aligned}$$

and let $\star _-$ denote the restriction of $\star$ to

$$\begin{aligned}{\mathbf {M}}_4^-{:}{=} \{\alpha \in {\mathbf {M}}_4: {\mathbf {N}}(\alpha )=-1\}.\end{aligned}$$

Recalling that $S_\mu = \{p\in S: p \equiv \mu 1 \bmod 4{\mathbb {Z}}\}$ and $R = \{p\in S: {{\,\mathrm{spin}\,}}({\mathfrak {p}},\sigma ) {{\,\mathrm{spin}\,}}({\mathfrak {p}},\sigma ^{-1})=1 \text { for all } \sigma \ne 1 \in {{\,\mathrm{Gal}\,}}(K/{\mathbb {Q}})\},$ for a fixed sign $\mu$, define $R_\mu {:}{=} R\cap S_\mu$.

### Theorem 4

Assume *K* satisfies properties (C1)-(C4). Then

$$\begin{aligned} d(R|S)= & {} \frac{\#\ker (\star )}{2^n}, \\ d(R_+|S_+)= & {} \frac{\#\ker (\star _+)}{2^{n-1}} \quad \text {and} \quad d(R_-|S_-) = \frac{\#\ker (\star _-)}{2^{n-1}}. \end{aligned}$$

### Proof

That $d(R|S) = \#\ker (\star )/2^n$ is proven in [9, Theorem 6.2], though it will also follow from the proof that $d(R_\mu |S_\mu ) = {\# \ker (\star _\mu )}/{ 2^{n-1}}$ since $d(S_\mu |S)=1/2$ and $\ker (\star )$ is the disjoint union of $\ker (\star _+)$ and $\ker (\star _-)$ and *R* is the disjoint union of $R_+$ and $R_-$.

Recall the map ${\mathbf {r}}_4$ from Definition 5. As shown in Definition 7, $\star (\alpha )=\star (\alpha ^\sigma )$ for any $\sigma \in {{\,\mathrm{Gal}\,}}(K/{\mathbb {Q}})$ so $\star \circ {\mathbf {r}}_4({\mathfrak {p}})=\star \circ {\mathbf {r}}_4({\mathfrak {p}}^\sigma )$ for any $\sigma \in {{\,\mathrm{Gal}\,}}(K/{\mathbb {Q}})$. By Proposition 1, for each fixed sign $\mu$,

$$\begin{aligned} R_\mu = \{p\in S_\mu : \star \circ {\mathbf {r}}_4({\mathfrak {p}})=1 \hbox { for }{\mathfrak {p}}\text { a prime of} K \hbox {above} p\}. \end{aligned}$$

For $N\in {\mathbb {Z}}_+$, let $R_{\mu ,N}{:}{=} \{p\in R_{\mu }: p<N\}$ and $S_{\mu ,N}{:}{=} \{p\in S_{\mu }: p<N\}$. We will prove that

$$\begin{aligned} d(R_\mu |S_\mu ) = \frac{\# \ker (\star _\mu )}{ \# {\mathbf {M}}_4^\mu }. \end{aligned}$$

Then since *K* is cyclic of odd degree and 2 is inert in $K/{\mathbb {Q}}$, we can apply Proposition 4 to get that $\#{\mathbf {M}}_4= 2^n$. Then since half the elements of ${\mathbf {M}}_4$ are in ${\mathbf {M}}_4^+$ and half in ${\mathbf {M}}_4^-$, $\#{\mathbf {M}}_4^+=\#{\mathbf {M}}_4^- = 2^{n-1}$.

Let $\mu$ denote a fixed sign. Let $S_{\mu ,N}'$ denote the set of primes of *K* laying above primes in $S_{\mu ,N}$ and let $R_{\mu ,N}'$ denote the set of primes of *K* laying above primes in $R_{\mu ,N}$. Since primes in *S* split completely,

$$\begin{aligned} \frac{\#R_{\mu ,N}}{\#S_{\mu ,N}}= \frac{\#R_{\mu ,N}'}{\#S_{\mu ,N}'}. \end{aligned}$$

Let ${\mathbf {r}}_{4,N}$ denote the restriction of ${\mathbf {r}}_4$ to $S_{\mu , N}'$. Then $R_{\mu ,N}'$ is the disjoint union

$$\begin{aligned} R_{\mu ,N}' = \bigsqcup _{\alpha \in \ker (\star _\mu )} \left( S_{\mu , N}' \cap {\mathbf {r}}_{4,N}^{-1}(\alpha )\right) , \end{aligned}$$

taken over elements $\alpha \in \ker (\star _\mu )$, i.e. elements of $\alpha \in {\mathbf {M}}_4$ such that ${\mathbf {N}}(\alpha )=\mu 1 \bmod 4$ and $\star (\alpha )=1$. Therefore

$$\begin{aligned} \frac{\#R_{\mu ,N}'}{\#S_{\mu ,N}'} = \sum _{\alpha \in \ker (\star _\mu )} \frac{\# \left( S_{\mu , N}' \cap {\mathbf {r}}_{4,N}^{-1}(\alpha )\right) }{\#S_{\mu ,N}'} \end{aligned}$$

By Lemma 9, for all $\alpha \in \ker (\star _\mu )$,

$$\begin{aligned} d({\mathbf {r}}_4^{-1}(\alpha )\cap S_{\mu }' | S_{\mu }') = \frac{1}{\#{\mathbf {M}}_4^\mu } = \frac{1}{2^{n-1}}. \end{aligned}$$

Therefore

$$\begin{aligned} d(R_\mu |S_\mu ) = d(R_\mu '|S_\mu ')&= \lim _{N\rightarrow \infty } \sum _{\alpha \in \ker (\star _\mu )} \frac{\# \left( S_{\mu , N}' \cap {\mathbf {r}}_{4,N}^{-1}(\alpha )\right) }{\#S_{\mu ,N}'}\\&=\sum _{\alpha \in \ker (\star _\mu )} \lim _{N\rightarrow \infty } \frac{\# \left( S_{\mu , N}' \cap {\mathbf {r}}_{4,N}^{-1}(\alpha )\right) }{\#S_{\mu ,N}'}\\&= \sum _{\alpha \in \ker (\star _\mu )} d({\mathbf {r}}_4^{-1}(\alpha )\cap S_{\mu }' | S_{\mu }')\\&= \sum _{\alpha \in \ker (\star _\mu )} \frac{1}{2^{n-1}} =\frac{\#\ker (\star _\mu ) }{2^{n-1}}. \end{aligned}$$

$$\square $$

## Counting solutions to a Hilbert symbol condition

In this section, we assume that $K/{\mathbb {Q}}$ satisfies (C1)-(C4) and prove formulae for $\#\ker (\star _{\mu })$. Throughout, the degree $n:=[K:{\mathbb {Q}}]$ to taken as an odd integer.

Fix $\tau$ to be a generator of ${{\,\mathrm{Gal}\,}}(K/{\mathbb {Q}})$. For any $\alpha \in K$, write $\alpha _{(k)}{:}{=} \alpha ^{\tau ^k}$ for $k\in {\mathbb {Z}}$.

### Lemma 10

$$(-1,-1)_2=-1$$.

### Proof

Assume for contradiction that $(-1,1)_2=1$. Consider a homomorphism $\psi :{\mathbf {M}}_4\rightarrow \{\pm 1\}$ given by $[\alpha ]\mapsto (\alpha ,-1)_2$. Since the Hilbert symbol is non-degenerate, and $-1$ is not a square modulo 4 in *K*, $\psi$ is not identically 1. Therefore $\#\ker \psi =\#{\mathbf {M}}_4/\#{\text {im}}\psi =2^{n-1}$.

For any $[\alpha ]\in {\mathbf {M}}_4\setminus \{\pm 1\}$, we have $(\alpha _{(k)},-1)_2=(\alpha ,-1)_2$ for any *k*. Therefore $\psi$ is stable under the Galois action. The size of each Galois orbit is *n* except the orbit of $\pm 1$. But then *n* divides both $\#\{[\alpha ]\in {\mathbf {M}}_4\setminus \{\pm 1\} :\psi (\alpha )=1\}=\#\{[\alpha ]\in {\mathbf {M}}_4 :\psi (\alpha )=1\}-2=2^{n-1}-2$ and $\#\{[\alpha ]\in {\mathbf {M}}_4 :\psi (\alpha )=-1\}=2^{n-1}$, which is a contradiction. $\square$

Our aim is to count the number of elements in ${\mathbf {M}}_4$ with a representative $\alpha \in {\mathcal {O}}_K$ satisfying the spin relation

6 $$\begin{aligned} (\alpha ,\alpha ^\sigma )_2=1 \text { for all non-trivial }\sigma \in {{\,\mathrm{Gal}\,}}(K/{\mathbb {Q}}). \end{aligned}$$

By Lemma 6, the property (6) only depends on the class of $[\alpha ]\in {\mathbf {M}}_4$.

### The Hilbert symbol as a bilinear form on ${\mathbf {M}}_4$

By the Kronecker–Weber theorem, *K* is contained in the cyclotomic field ${\mathbb {Q}}(\zeta _{{\mathfrak {f}}})$, where ${\mathfrak {f}}$ is the conductor of *K*. The conductor ${\mathfrak {f}}$ is odd since we assumed that 2 is unramified in *K*. By [4, Theorem 4.5], there exists a normal 2-integral basis of ${\mathbb {Q}}(\zeta _{{\mathfrak {f}}})$, i.e. we can find some $a\in {\mathcal {O}}_{{\mathbb {Q}}(\zeta _{{\mathfrak {f}}})}$ such that the localization of ${\mathcal {O}}_{{\mathbb {Q}}(\zeta _{{\mathfrak {f}}})}$ at 2 can be written as ${\mathcal {O}}_{{\mathbb {Q}}(\zeta _{{\mathfrak {f}}}),2}=\oplus _{g\in {{\,\mathrm{Gal}\,}}({\mathbb {Q}}(\zeta _{{\mathfrak {f}}})/{\mathbb {Q}})}{\mathbb {Z}}_{(2)}a^{g}$. Similar to the classic result for integral bases [11, Proposition 4.31(i)], taking $y={{\,\mathrm{Tr}\,}}_{{\mathbb {Q}}(\zeta _{{\mathfrak {f}}})/K} (a)$, then $\{y,y^\tau , \dots , y^{\tau ^{n-1}}\}$ gives a normal 2-integral basis of *K*. Since ${\mathbb {Z}}_{(2)}/2\cong {\mathbb {Z}}/2$ and ${\mathcal {O}}_{K,2}/2\cong {\mathcal {O}}_K/2$, we know that $y,y^\tau , \dots , y^{\tau ^{n-1}}$ also form a normal ${\mathbb {F}}_2$-basis of ${\mathcal {O}}_K/2$.

Set $\alpha =1+2y$. It follows from the isomorphism in (5) that

$$\begin{aligned}{\mathbf {M}}_4=\left\{ \prod _{i=0}^{n-1}[\alpha _{(i)}]^{u_i}:(u_0,\dots ,u_{n-1})\in {\mathbb {F}}_2^n\right\} .\end{aligned}$$

Write $(\alpha ,\alpha _{(i)})_2=(-1)^{c_i}$, $c_i\in \{0,1\}$. Note that $(\alpha _{(i)},\alpha _{(j)})_2=(\alpha ,\alpha _{(j-i)})_2$. The Hilbert symbol is multiplicatively bilinear, so we can represent $(\text{[ }1.25ex]\mathbf{\cdot },\text{[ }1.25ex]\mathbf{\cdot })_2$ by the matrix

7 $$\begin{aligned} A{:}{=} \begin{pmatrix} c_0 &{} c_{n-1} &{} c_{n-2} &{} \dots &{} c_1 \\ c_1 &{} c_0 &{} c_{n-1} &{} \dots &{} c_2 \\ c_2 &{} c_1 &{} c_0 &{} \dots &{} c_3 \\ \vdots &{} \vdots &{} \vdots &{} \ddots &{} \vdots \\ c_{n-1} &{} c_{n-2} &{} c_{n-3} &{} \dots &{} c_0 \\ \end{pmatrix} \end{aligned}$$

with respect to the basis $[\alpha _{(i)}]$, $0\le i\le n-1$.

Define the $n\times n$ ${\mathbb {F}}_2$-matrix

$$\begin{aligned}T_1= \begin{pmatrix} 0 &{}\quad 1 &{}\quad 0 &{}\quad 0 &{}\quad \dots &{}\quad 0 \\ 0 &{}\quad 0 &{}\quad 1 &{}\quad 0 &{}\quad \dots &{}\quad 0 \\ 0 &{}\quad 0 &{}\quad 0 &{}\quad 1 &{}\quad \dots &{}\quad 0 \\ \vdots &{}\quad \vdots &{}\quad \vdots &{}\quad \vdots &{}\quad \ddots &{}\quad \vdots \\ 0 &{}\quad 0 &{}\quad 0 &{}\quad 0 &{}\quad \dots &{}\quad 1 \\ 1 &{}\quad 0 &{}\quad 0 &{}\quad 0 &{}\quad \dots &{}\quad 0 \\ \end{pmatrix}, \end{aligned}$$

$$T_k=T_1^k$$ and $$T_0=I$$.

#### Lemma 11

Let *A* be the matrix representation of $(\text{[ }1.25ex]\mathbf{\cdot },\text{[ }1.25ex]\mathbf{\cdot })_2$ on ${\mathbf {M}}_4$ with respect to a normal basis, as given in (7). Define a map

$$\begin{aligned} \Psi :\&{\mathbb {F}}_2^n\rightarrow {\mathbb {F}}_2[x]/(x^n-1)\\&{\mathbf {u}}=(u_0,\dots ,u_{n-1}) \mapsto F_{{\mathbf {u}}}(x){:}{=} u_0+u_1x+u_2x^2+\dots +u_{n-1}x^{n-1}. \end{aligned}$$

Also define

$$\begin{aligned} \Phi : {\mathbb {F}}_2^n \rightarrow {\mathbb {F}}_2^n \qquad {\mathbf {u}} \mapsto ( {\mathbf {u}}^T T_0{\mathbf {u}},\ {\mathbf {u}}^T T_1{\mathbf {u}},\ \dots ,\ {\mathbf {u}}^T T_{n-1}{\mathbf {u}} ). \end{aligned}$$

Let $$B{:}{=}\Psi \circ \Phi $$, so

$$\begin{aligned} B: {\mathbb {F}}_2^n \rightarrow {\mathbb {F}}_2[x]/(x^n-1) \qquad {\mathbf {u}} \mapsto x^n\cdot F_{{\mathbf {u}}}(x)F_{{\mathbf {u}}}(1/x)\bmod (x^n-1). \end{aligned}$$

Then $\#\ker (\star _+) =\#B^{-1}(0)$ and $\#\ker (\star _-) =\# B^{-1}(h(x))$, where $h(x)=\Psi (A^{-1}(1,0,\dots ,0))$. Furthermore

8 $$\begin{aligned} h(x)\equiv x^n h(1/x)\bmod (x^n-1). \end{aligned}$$

#### Proof

For any ${\mathbf {u}}=(u_0,\dots ,u_{n-1}), {\mathbf {v}}=(v_0,\dots ,v_{n-1})\in {\mathbb {F}}_2^{n}$, we have

$$\begin{aligned}\left( \prod _{i}\alpha _{(i)}^{u_i},\prod _{j}\alpha _{(j)}^{v_j}\right) _2 =(-1)^{{\mathbf {u}}^TA{\mathbf {v}}}.\end{aligned}$$

Since $(\text{[ }1.25ex]\mathbf{\cdot },\text{[ }1.25ex]\mathbf{\cdot })_2$ is non-degenerate on ${\mathbf {M}}_4$ by Lemma 6, the matrix *A* has rank *n* and is invertible. Note also that *A* is symmetric.

Now $\prod _{i}\alpha _{(i)}^{u_i}$, ${\mathbf {u}}=(u_0,\dots ,u_{n-1})\in {\mathbb {F}}_2^{n}$ satisfies (6) if and only if

9 $$\begin{aligned} {\mathbf {u}}^TAT_1{\mathbf {u}}={\mathbf {u}}^TAT_2{\mathbf {u}}=\dots ={\mathbf {u}}^TAT_{n-1}{\mathbf {u}}=0. \end{aligned}$$

Notice that from (7), we can write

$$\begin{aligned}A=\sum _{i=0}^{n-1}c_iT_i,\qquad c_i\in {\mathbb {F}}_2.\end{aligned}$$

Then (9) becomes

10 $$\begin{aligned} A\circ \Phi ({\mathbf {u}})=A \begin{pmatrix} {\mathbf {u}}^T T_0{\mathbf {u}} \\ {\mathbf {u}}^T T_1{\mathbf {u}} \\ \vdots \\ {\mathbf {u}}^T T_{n-1}{\mathbf {u}} \\ \end{pmatrix} \in \left\{ \begin{pmatrix} 0 \\ 0 \\ \vdots \\ 0 \\ \end{pmatrix}, \begin{pmatrix} 1 \\ 0 \\ \vdots \\ 0 \\ \end{pmatrix} \right\} . \end{aligned}$$

Since *A* is invertible, we can set $h(x)=\Psi (A^{-1}(1,0,\dots ,0))$. Notice that $\Psi$ is a one-to-one correspondence. Then (10) can be rewritten as $B({\mathbf {u}})=\Psi \circ \Phi ({\mathbf {u}})\in \{0,h(x)\}$. Since *A* is symmetric, $A^{-1}$ is also symmetric, so (8) holds. Also $(\alpha ,\alpha )_2=(\alpha ,-1)_2=(\alpha ,-1)_2^n=\prod _{i}(\alpha _{(i)},-1)_2=({\mathfrak {N}}_{K/{\mathbb {Q}}}(\alpha ),-1)_2$, which is 1 if ${\mathfrak {N}}_{K/{\mathbb {Q}}}(\alpha )\equiv 1\bmod 4$ and $-1$ if ${\mathfrak {N}}_{K/{\mathbb {Q}}}(\alpha )\equiv -1\bmod 4$ by Lemma 10. Therefore $\#\ker (\star _+) =\#B^{-1}(0)$ and $\#\ker (\star _-) =\# B^{-1}(h(x))$. $\square$

### The counting problem

Our aim is to obtain the size of the preimage of 0 and *h*(*x*) under *B*. For any polynomial *f*, let $f^*$ denote its reciprocal, i.e. $f^*(x)=x^{\deg f}\cdot f(1/x)$.

#### Lemma 12

For any positive factor $k\ne 1$ of *n*, let $d_k$ be the order of 2 in $({\mathbb {Z}}/k)^\times$. Also set $d_1=1$. Consider the following factorisation in ${\mathbb {F}}_2[x]$,

$$\begin{aligned} x^n-1= f_1(x)\dots f_r(x)f^*_{m+1}(x)\dots f^*_r(x), \end{aligned}$$

where $f_i$ are irreducible and $f_i=f_i^*$ for $i=1,\dots , m$. Let $m_k$ be the number of *i* such that $f_i=f_i^*$ and $\deg f_i=d_k$ and let $2r_k-m_k$ be the number of *i* such that $\deg f_i=d_k$. Then $\sum _{i=1}^r\deg f_i=\sum _{k\mid n}r_kd_k$ and $r=\sum _{k\mid n}r_k$ and $m=\sum _{k\mid n}m_k$, where $r_1=m_1=1$, and

$$\begin{aligned}(r_k,m_k)= {\left\{ \begin{array}{ll} \quad \left( \frac{\phi (k)}{2d_k},\ 0\right) &{} \text { if }d_k\text { is odd,}\\ \left( \frac{\phi (k)}{d_k},\ \frac{\phi (k)}{d_k}\right) &{} \text { if }d_k\text { is even,} \end{array}\right. }\end{aligned}$$

for $$k\ne 1$$.

#### Proof

Take *f* to be an irreducible factor of $x^n-1$ in ${\mathbb {F}}_2[x]$. Let $\gamma$ be a root of *f* in an extension of ${\mathbb {F}}_2$. Then $\gamma$ is a primitive *k*-th root of unity, where *k* is some integer dividing *n*. Galois theory on finite fields shows that ${{\,\mathrm{Gal}\,}}({\mathbb {F}}_2(\gamma )/{\mathbb {F}}_2)$ is generated by the Frobenius $\varphi : x\mapsto x^2$. Since $\varphi ^i: x\mapsto x^{2^i}$ for any $i\in {\mathbb {Z}}$, we see that the order of $\varphi$ must be $d_k$, the order of 2 in $({\mathbb {Z}}/k)^{\times }$. Therefore $\deg f=d_k$. The set of roots of *f* is $\{\gamma , \varphi (\gamma ),\varphi ^2(\gamma ),\dots , \varphi ^{d_k-1}(\gamma )\}$, which is closed under inversion precisely when $d_k$ is even. Therefore *f* is self-reciprocal if and only if $d_k$ is even.

Let $A_k$ be the set of distinct irreducible factors of $x^n-1$ in ${\mathbb {F}}_2[x]$ which has a primitive *k*-th root of unity in an extension of ${\mathbb {F}}_2$, and $M_k$ be a subset of $A_k$ containing elements which are self-reciprocal, so $2r_k-m_k=\#A_k$ and $m_k=\#M_k$. If $d_k$ is even, then all $f\in A_k$ are self-reciprocals, so $A_k=M_k$ and $r_k=m_k$. If $d_k$ is odd, then $M_k=\varnothing$ and $m_k=0$.

There are $\phi (k)$ roots of $x^n-1$ which are primitive *k*-th root of unity, so $(2r_k-m_k)d_k=\phi (k)$. Now the statement of the Lemma follows from $r_k=m_k$ when $d_k$ is even, and $m_k=0$ when $d_k$ is odd. $\square$

We are now ready to prove the formulae for $\#\ker (\star _+)$ and $\#\ker (\star _-)$.

#### Proposition 5

For each $k\ne 1$ dividing *n*, let $d_k$ be the order of 2 in $({\mathbb {Z}}/k)^\times$. Then

$$\begin{aligned}\#\ker (\star _+)=\prod _{k\mid n,\ d_k\text {odd},\ k\ne 1}(2^{1+d_k}-1)^{\frac{\phi (k)}{2d_k}},\end{aligned}$$

and

$$\begin{aligned}\#\ker (\star _-)=\prod _{k\mid n,\ d_k\text {even},\ k\ne 1}(2^{d_k/2}+1)^{\frac{\phi (k)}{d_k}}\prod _{k\mid n,\ d_k\text {odd},\ k\ne 1}(2^{d_k}-1)^{\frac{\phi (k)}{2d_k}}.\end{aligned}$$

If *n* is a prime, then writing $d=d_n$,

$$\begin{aligned} (\#\ker (\star _+),\#\ker (\star _-)) ={\left\{ \begin{array}{ll} \left( (2^{1+d}-1)^{\frac{n-1}{2d}},\ (2^d-1)^{\frac{n-1}{2d}}\right) &{} \text { if }d\text { is odd,}\\ \quad \left( 1,\ (2^{\frac{d}{2}}+1)^{\frac{n-1}{d}}\right) &{} \text { if }d\text { is even.} \end{array}\right. } \end{aligned}$$

In particular, when $n=3$, $\#\ker (\star _+)=1$ and $\#\ker (\star _-) =3$.

#### Proof

The first case we make use of $\#\ker (\star _+) =\#B^{-1}(0)$ from Lemma 11. Here $B({\mathbf {u}})=0$ implies $(x^n-1)\mid F_{{\mathbf {u}}}(x)F^*_{{\mathbf {u}}}(x)$. Obtain the following factorisation in ${\mathbb {F}}_2[x]$ as described in Lemma 12,

11 $$\begin{aligned} x^n-1= f_1(x)\dots f_r(x)f^*_{m+1}(x)\dots f^*_r(x), \end{aligned}$$

Then for each $k=1,\dots , r$, we have $f_k\mid F_{{\mathbf {u}}}$ or $f_k^*\mid F_{{\mathbf {u}}}$.

By the Chinese Remainder Theorem,

$$\begin{aligned}{\mathbb {F}}_2[x]/(x^n-1) \cong \prod _{i=1}^{r}\left( {\mathbb {F}}_2[x]/(f_i)\right) \times \prod _{j={m+1}}^{r}\left( {\mathbb {F}}_2[x]/(f^*_{j})\right) .\end{aligned}$$

For $k=1,\dots ,m$, the image of $F_{{\mathbf {u}}}$ in ${\mathbb {F}}_2[x]/(f_k)$ is 0. For $k=m+1,\dots r$, the image of $F_{{\mathbf {u}}}$ is 0 in at least one of ${\mathbb {F}}_2[x]/(f_k)$ and ${\mathbb {F}}_2[x]/(f^*_k)$. Therefore

$$\begin{aligned}\#B^{-1}(0)=\prod _{j={m+1}}^{r}(2^{1+\deg f_j}-1).\end{aligned}$$

Now applying Lemma 12,

12 $$\begin{aligned} \#\ker (\star _+)=\prod _{k\mid n}(2^{1+d_k}-1)^{r_k-m_k} =\prod _{k\mid n,\ d_k\text { odd},\ k\ne 1}(2^{1+d_k}-1)^{\frac{\phi (k)}{2d_k}} .\end{aligned}$$

The second case $\#\ker (\star _-)$ we consider $B({\mathbf {u}})=h(x)$. We count the number of ${\mathbf {u}}\in {\mathbb {F}}_2^n$ such that

13 $$\begin{aligned} x^n\cdot F_{{\mathbf {u}}}(x)F_{{\mathbf {u}}}(1/x)\equiv h(x)\bmod (x^n-1). \end{aligned}$$

Since *A* has full rank and $(1,0,0,\dots 0),(0,1,0,\dots , 0),(0,0,\dots ,0, 1)$ are linearly independent, we know that $h(x),xh(x), \cdots , x^{n-1}h(x)$ are linearly independent in ${\mathbb {F}}_2[x]/(x^n-1)$. This implies that $h(x)\in ({\mathbb {F}}_2[x]/(x^n-1))^\times$.

Fix a primitive complex *n*-th root of unity $\zeta _n$. Consider the isomorphism

$$\begin{aligned} ({\mathbb {F}}_2[x]/(x^n-1))^{\times }\rightarrow ({\mathbb {Z}}[\zeta _n]/2)^{\times }&F_{{\mathbf {u}}}(x) \mapsto F_{{\mathbf {u}}}(\zeta _n)\bmod 2. \end{aligned}$$

Now (13) becomes

$$\begin{aligned}F_{{\mathbf {u}}}(\zeta _n)\overline{F_{{\mathbf {u}}}(\zeta _n)}\equiv h(\zeta _n)\bmod 2.\end{aligned}$$

Notice from (8) that $h(\zeta _n)=h(\zeta _n^{-1})=\overline{h(\zeta _n)}$ is real. We compute from (11),

$$\begin{aligned}&\#({\mathbb {Z}}[\zeta _n]/2)^{\times } =\#({\mathbb {F}}_2[x]/(x^n-1))^{\times }\\&\quad =\prod _{i=1}^{r}\#\left( {\mathbb {F}}_2[x]/(f_i)\right) ^{\times } \prod _{j={m+1}}^{r}\#\left( {\mathbb {F}}_2[x]/(f^*_{j})\right) ^{\times } =\prod _{k\mid n}(2^{d_k}-1)^{2r_k-m_k}.\end{aligned}$$

Take $g\in {\mathbb {F}}_2[x]$ such that

$$\begin{aligned}\frac{x^n-1}{x-1}\equiv x^{n-1}+x^{n-2}+\dots +x+1= x^{\frac{n-1}{2}}g(x+x^{-1}).\end{aligned}$$

We can factorise $g(x)=g_2(x)\dots g_r(x)$, where $x^{\deg g_k}\cdot g_k(x+x^{-1})=f_k(x)$ for $2\le k\le m$ and $x^{\deg g_k}\cdot g_k(x+x^{-1})=f_k(x)f_k^*(x)$ for $m+1\le k\le r$. Then since $({\mathbb {Z}}[\zeta _n+\zeta _n^{-1}]/2)^{\times }\cong ({\mathbb {F}}_2[x]/(g))^{\times }$, we compute

$$\begin{aligned}&\#\left( {\mathbb {Z}}[\zeta _n+\zeta _n^{-1}]/2\right) ^{\times } =\#({\mathbb {F}}_2[x]/(g))^{\times }\\&\quad =\prod _{i=2}^{r}\#\left( {\mathbb {F}}_2[x]/(g_i)\right) ^{\times } =\prod _{k\mid n,\ k\ne 1}(2^{d_k/2}-1)^{m_k}(2^{d_k}-1)^{r_k-m_k}. \end{aligned}$$

Our goal is to compute the size of the kernel of the homomorphism

$$\begin{aligned} \psi :({\mathbb {Z}}[\zeta _n]/2)^{\times }\rightarrow ({\mathbb {Z}}[\zeta _n+\zeta _n^{-1}]/2)^{\times } \beta \mapsto \beta {\overline{\beta }}. \end{aligned}$$

We claim that $\psi$ is surjective. Since $({\mathbb {Z}}[\zeta _n+\zeta _n^{-1}]/2)^{\times }$ has odd order, every element is a square, so suppose $\beta ^2\in ({\mathbb {Z}}[\zeta _n+\zeta _n^{-1}]/2)^{\times }$, then $\psi ({\hat{\beta }})=\beta ^2$ for any lift ${\hat{\beta }}\in {\mathbb {Z}}[\zeta _n+\zeta _n^{-1}]$ of $\beta$. Therefore

14 $$\begin{aligned}&\#\ker (\star _-) =\# B^{-1}(h(x)) =\#\ker \psi \nonumber \\&=\frac{\#({\mathbb {Z}}[\zeta _n]/2)^{\times }}{\#{\text {im}}\psi } =\prod _{k\mid n,\ k\ne 1}(2^{d_k/2}+1)^{m_k}(2^{d_k}-1)^{r_k-m_k}. \end{aligned}$$

Putting in (12) and (14) the values of *r* and *m* in terms of *n* and *d* as in Lemma 12 proves the proposition. $\square$

## Joint spins

Fix a sign $\mu \in \{\pm \}$. Recall that $S_{\mu }$ is the set of rational primes $p\equiv \mu 1 \bmod 4$ that split completely in $K/{\mathbb {Q}}$, i.e., unramified and of residue degree 1 in $K/{\mathbb {Q}}$, and that $F_{\mu }$ is the set of $p\in S_{\mu }$ of residue degree 1 in $K(p)/{\mathbb {Q}}$. By Corollary 1, a prime $p\in S_{\mu }$ belongs to $F_{\mu }$ if and only if ${{\,\mathrm{spin}\,}}({\mathfrak {p}}, \sigma ) = 1$ for all non-trivial $\sigma \in {{\,\mathrm{Gal}\,}}(K/{\mathbb {Q}})$ and any prime ideal ${\mathfrak {p}}$ of *K* lying above *p*. Recall that $R_{\mu }$ is the set of primes $p\in S_{\mu }$ such that ${{\,\mathrm{spin}\,}}({\mathfrak {p}}, \sigma ){{\,\mathrm{spin}\,}}({\mathfrak {p}}, \sigma ^{-1}) = 1$ for all non-trivial $\sigma \in {{\,\mathrm{Gal}\,}}(K/{\mathbb {Q}})$ and all prime ideals ${\mathfrak {p}}$ of *K* lying above *p*, so that $F_{\mu }\subset R_{\mu }$. In this section, we will prove the following formula for the relative density of $F_{\mu }$ in $R_{\mu }$, denoted by $d(F_{\mu }|R_{\mu })$.

### Theorem 5

Assume Conjecture $C_{\eta }$ for $\eta = \frac{2}{n(n-1)}$. Then

$$\begin{aligned} d(F_{\mu }|R_{\mu }) = 2^{-\frac{n-1}{2}}. \end{aligned}$$

Since each $p\in S_{\mu }$ splits into exactly the same number of prime ideals in ${\mathcal {O}}$, and since $R_{\mu }$ is a set of primes of positive natural density, it suffices to show that

15 $$\begin{aligned} \sum _{\begin{array}{c} {\mathfrak {N}}({\mathfrak {p}})\le X \\ {\mathfrak {p}}\text { lies over }p\in F_{\mu } \end{array}}1 = 2^{-\frac{n-1}{2}}\sum _{\begin{array}{c} {\mathfrak {N}}({\mathfrak {p}})\le X \\ {\mathfrak {p}}\text { lies over }p\in R_{\mu } \end{array}}1 + o(X(\log X)^{-1}). \end{aligned}$$

Let $\tau$ be a generator of ${{\,\mathrm{Gal}\,}}(K/{\mathbb {Q}})$, a cyclic group of order *n*. Then, by definition of the set $R_{\mu }$, a prime $p\in R_{\mu }$ belongs to the set $F_{\mu }$ if and only if ${{\,\mathrm{spin}\,}}({\mathfrak {p}},\tau ^k)=1$ for all $k\in \{1, 2, \ldots , \frac{n-1}{2}\}$. The product

$$\begin{aligned} \prod _{k = 1}^{\frac{n-1}{2}}\frac{1+{{\,\mathrm{spin}\,}}({\mathfrak {p}}, \tau ^k)}{2} \end{aligned}$$

is the indicator function of the property that ${{\,\mathrm{spin}\,}}({\mathfrak {p}}, \tau ^k) = 1$ for all $k\in \{1, 2,\ldots , \frac{n-1}{2}\}$. Expanding this product gives

16 $$\begin{aligned} 2^{-\frac{n-1}{2}}\sum _{H\subset \{\tau , \ldots , \tau ^{\frac{n-1}{2}}\}}\prod _{\sigma \in H}{{\,\mathrm{spin}\,}}({\mathfrak {p}}, \sigma ), \end{aligned}$$

where the sum is over all subsets *H* of $\{\tau , \tau ^2, \ldots , \tau ^{\frac{n-1}{2}}\}$. When $H = \emptyset$, the product is 1 by convention.

Let *L*/*K* be any abelian extension whose Galois group is isomorphic to ${\mathbf {M}}_4^{\mu }$, and let ${\mathcal {A}}$ denote the set of disjoint ${{\,\mathrm{Gal}\,}}(K/{\mathbb {Q}})$-orbits of elements of ${\mathbf {M}}_4^{\mu }$, so that we can write

$$\begin{aligned} {\mathbf {M}}_4^{\mu } = \bigsqcup _{A\in {\mathcal {A}}} A. \end{aligned}$$

Each ${{\,\mathrm{Gal}\,}}(K/{\mathbb {Q}})$-orbit *A* is then a collection of invertible congruence classes modulo $4{\mathcal {O}}$ that are distinct modulo squares. Let ${\mathcal {A}}_0\subset {\mathcal {A}}$ be the set of ${{\,\mathrm{Gal}\,}}(K/{\mathbb {Q}})$-orbits *A* such that ${{\,\mathrm{spin}\,}}({\mathfrak {p}}, \sigma ){{\,\mathrm{spin}\,}}({\mathfrak {p}}, \sigma ^{-1})=1$ for all non-trivial $\sigma \in G$ and for all prime ideals ${\mathfrak {p}}$ such that ${\mathbf {r}}_4({\mathfrak {p}})\in A$. Note that a prime ideal ${\mathfrak {p}}$ in ${\mathcal {O}}$ lies over a prime $p\in R_{\mu }$ if and only if ${\mathbf {r}}_4({\mathfrak {p}}) \in A$ for some $A\in {\mathcal {A}}_0$.

Summing (16) over all prime ideals ${\mathfrak {p}}$ of norm ${\mathfrak {N}}({\mathfrak {p}})\le X$, we get that

$$\begin{aligned} \sum _{\begin{array}{c} {\mathfrak {N}}({\mathfrak {p}})\le X \\ p\in F_{\mu } \end{array}}1 = 2^{-\frac{n-1}{2}}\sum _{\begin{array}{c} H\subset \{\tau , \ldots , \tau ^{\frac{n-1}{2}}\} \\ A\in {\mathcal {A}}_0 \end{array}}\Sigma (X; H, A), \end{aligned}$$

where

$$\begin{aligned} \Sigma (X; H, A) = \sum _{\begin{array}{c} {\mathfrak {N}}({\mathfrak {p}})\le X \\ {\mathbf {r}}_4({\mathfrak {p}})\in A \end{array}}\prod _{\sigma \in H}{{\,\mathrm{spin}\,}}({\mathfrak {p}}, \sigma ). \end{aligned}$$

Being able to split the sum of interest into sums of the type $\Sigma (X; H, A)$ as above is what partially motivates introducing *L*/*K* and orbits *A*, as it is unclear how one could cleanly define an analogue of $\Sigma (X; H, A)$ for just one congruence class modulo $4{\mathcal {O}}$ at a time (as opposed to one orbit *A*).

The sums $\Sigma (X; \emptyset , A)$ feature no cancellation and provide the main term in (15). It then remains to show that

17 $$\begin{aligned} \Sigma (X; H, A) = o(X/\log X) \end{aligned}$$

for each non-empty subset *H* of $\{\tau , \ldots , \tau ^{\frac{n-1}{2}}\}$ and each $A\in {\mathcal {A}}_0$. To this end, we will use a slight generalization of Theorem 1 of [6].

We cannot apply the results of [6] directly for two reasons. First, the class number *h* of *K* need not be 1 – this forces us to relate ${{\,\mathrm{spin}\,}}({\mathfrak {a}}, \sigma )$ to quadratic residue symbols involving elements “smaller” than the totally positive generators of ${\mathfrak {a}}^h$. Second, the sums $\Sigma (X; H, A)$ feature the additional restriction that ${\mathbf {r}}_4({\mathfrak {p}})\in A$. Since *A* is a collection of congruence classes modulo $4{\mathcal {O}}$, the restriction that ${\mathbf {r}}_4({\mathfrak {p}})\in A$ is reminiscent of the restriction to a congruence class as in [5, Theorem 1.2, p. 699]. Despite the similarity, there is a technical difference that we will explain.

Fix once and for all a set ${\mathcal {C}}$ consisting of *h* unramified degree-one prime ideals in ${\mathcal {O}}$ that is a complete set of representatives of ideal classes in the class group of *K*; its existence is guaranteed by an application of the Chebotarev Density Theorem to the Hilbert class field of *K*.

Now suppose that ${\mathfrak {a}}$ is a non-zero ideal in ${\mathcal {O}}$ coprime to $\prod _{{\mathfrak {p}}\in {\mathcal {C}}}{\mathfrak {N}}({\mathfrak {p}})$, and let $\alpha$ denote a totally positive generator of ${\mathfrak {a}}^h$. As *h* is odd, the set $\{{\mathfrak {p}}^2: {\mathfrak {p}}\in {\mathcal {C}}\}$ is also a complete set of representatives. Hence there exists ${\mathfrak {p}}\in {\mathcal {C}}$ such that ${\mathfrak {a}}{\mathfrak {p}}^2$ is a principal ideal. Let $\pi$ denote a totally positive generator of the ideal ${\mathfrak {p}}^h$. Let $\alpha _0$ denote a totally positive generator of ${\mathfrak {a}}{\mathfrak {p}}^2$. Then $\alpha _0^h$ and $\alpha \pi ^2$ are both totally positive generators of the ideal $({\mathfrak {a}}{\mathfrak {p}}^{2})^h$, so we have

18 $$\begin{aligned} {{\,\mathrm{spin}\,}}({\mathfrak {a}}, \sigma ) = \genfrac(){}{}{\alpha }{\sigma ({\mathfrak {a}})} = \genfrac(){}{}{\alpha \pi ^2}{\sigma ({\mathfrak {a}}{\mathfrak {p}}^2)} = {{\,\mathrm{spin}\,}}({\mathfrak {a}}{\mathfrak {p}}^2, \sigma ) = \genfrac(){}{}{\alpha _0^h}{\sigma ({\mathfrak {a}}{\mathfrak {p}}^2)} = \genfrac(){}{}{\alpha _0}{\sigma (\alpha _0)}, \end{aligned}$$

since *h* is odd. Note that for each ${\mathfrak {p}}\in {\mathcal {C}}$ there is a bijection

19 $$\begin{aligned}&\{{\mathfrak {a}}\subset {\mathcal {O}}: {\mathfrak {N}}({\mathfrak {a}})\le x, {\mathfrak {a}}{\mathfrak {p}}^2\text { is principal}\} \nonumber \\&\quad \simeq \{\alpha _0\in {\mathcal {D}}: {\mathfrak {N}}(\alpha _0)\le x{\mathfrak {N}}({\mathfrak {p}})^2, \alpha _0\equiv 0\bmod {\mathfrak {p}}^2\} \end{aligned}$$

given by ${\mathfrak {a}}\mapsto \alpha _0$ as above, and where ${\mathcal {D}}$ is a set of totally positive elements in ${\mathcal {O}}$ defined in [5, (4.2), p.713]. Moreover, ${\mathbf {r}}_4({\mathfrak {a}})$ is the class in ${\mathbf {M}}_4$ of a totally positive generator of ${\mathfrak {a}}^h$, i.e., the class of $\alpha$ in ${\mathbf {M}}_4$. Since squares vanish in ${\mathbf {M}}_4$, the classes of $\alpha$ and $\alpha \pi ^2$, and so also of $\alpha _0^h$, coincide in ${\mathbf {M}}_4$. Hence, if *A* is a ${{\,\mathrm{Gal}\,}}(K/{\mathbb {Q}})$-orbit, then

20 $$\begin{aligned} {\mathbf {r}}_4({\mathfrak {a}})\in A\quad \text { if and only if }\quad \alpha _0^h\in A. \end{aligned}$$

We will now prove the following adaptation of [6, Theorem 1, p. 2].

### Theorem 6

With notation as above, let *H* be a non-empty subset of $\{\tau , \ldots , \tau ^{\frac{n-1}{2}}\}$. Assume Conjecture $C_{\eta }$ holds true for $\eta = 1/(|H|n)$ with $\delta = \delta (\eta ) > 0$ (see [6, p. 7]). Let $\epsilon > 0$ be a real number. Then for all $X\ge 2$, we have

$$\begin{aligned} \Sigma (X; H, A) \ll X^{1 - \frac{\delta }{54|H|^2n(12n+1)} + \epsilon }, \end{aligned}$$

where the implied constant depends only on $\epsilon$ and *K*.

Note that the set *H* above is of size at most $\frac{n-1}{2}$. Since Conjecture $C_{\eta _1}$ implies Conjecture $C_{\eta _2}$ whenever $\eta _1\le \eta _2$, we see that, conditional on Conjecture $C_{\eta }$ for $\eta = \frac{2}{n(n-1)}$, Theorem 6 implies (17) for each ${{\,\mathrm{Gal}\,}}(K/{\mathbb {Q}})$-orbit $A\in {\mathcal {A}}_0$ and each non-empty subset $H\subset \{\tau , \ldots , \tau ^{\frac{n-1}{2}}\}$, and hence also Theorem 5. It thus remains to prove Theorem 6.

For a non-zero ideal ${\mathfrak {a}}\subset {\mathcal {O}}$ and a ${{\,\mathrm{Gal}\,}}(K/{\mathbb {Q}})$-orbit *A*, let

$$\begin{aligned} r({\mathfrak {a}}; A) = {\left\{ \begin{array}{ll} 1 &{} \text {if }{\mathbf {r}}_4({\mathfrak {a}})\in A \\ 0 &{} \text {otherwise,} \end{array}\right. } \end{aligned}$$

and let

$$\begin{aligned} s_{{\mathfrak {a}}} = r({\mathfrak {a}}; A)\prod _{\sigma \in H}{{\,\mathrm{spin}\,}}({\mathfrak {a}}, \sigma ). \end{aligned}$$

Then we have

$$\begin{aligned} \Sigma (X; H, A) = \sum _{{\mathfrak {N}}({\mathfrak {p}})\le X} s_{{\mathfrak {p}}}, \end{aligned}$$

where the summation is over prime ideals ${\mathfrak {p}}\subset {\mathcal {O}}$ of norm at most *X*.

Let *F* be the integer defined in [6, (2.2), p. 5]; it depends only on *K*. Moreover, we can choose the sets ${\mathcal {C}}\ell _a$ and ${\mathcal {C}}\ell _b$ in [6, p. 5] so that their elements are coprime to $\prod _{{\mathfrak {p}}\in {\mathcal {C}}}{\mathfrak {N}}({\mathfrak {p}})$. Note that *F* is divisible by 32.

To deduce Theorem 6, it suffices to prove that

$$\begin{aligned} \sum _{\begin{array}{c} {\mathfrak {N}}({\mathfrak {p}})\le X \\ {\mathfrak {p}}\not \mid F \end{array}} s_{{\mathfrak {p}}} \ll _{\epsilon , K} X^{1 - \frac{\delta }{54|H|^2n(12n+1)} + \epsilon } \end{aligned}$$

because *F* has only finitely many prime ideal divisors.

The proof of Theorem 6 proceeds via Vinogradov’s method, with suitable estimates necessary for the sums of type I

$$\begin{aligned} A_{{\mathfrak {m}}}(x) = \sum _{\begin{array}{c} {\mathfrak {N}}{{\mathfrak {a}}} \le x \\ ({\mathfrak {a}}, F) = 1,\ {\mathfrak {m}} \mid {\mathfrak {a}} \end{array}} s_{{\mathfrak {a}}}, \end{aligned}$$

where ${\mathfrak {m}}$ is any non-zero ideal coprime to $\tau ({\mathfrak {m}})$, and sums of type II

$$\begin{aligned} B(x, y; v, w) = \sum _{\begin{array}{c} {\mathfrak {N}}({\mathfrak {a}})\le x\\ ({\mathfrak {a}}, F) = 1 \end{array}}\sum _{\begin{array}{c} {\mathfrak {N}}({\mathfrak {b}})\le y\\ ({\mathfrak {b}}, F) = 1 \end{array}}v_{{\mathfrak {a}}}w_{{\mathfrak {b}}}s_{{\mathfrak {a}}{\mathfrak {b}}}, \end{aligned}$$

where $v = \{v_{{\mathfrak {a}}}\}_{{\mathfrak {a}}}$ and $w = \{w_{{\mathfrak {b}}}\}_{{\mathfrak {b}}}$ are arbitrary sequences of complex numbers of modulus bounded by 1. By [5, Proposition 5.2, p. 722] applied with $\vartheta = \frac{\delta }{54n|H|^2}$ and $\theta = \frac{1}{6n}$, the following two propositions imply Theorem 6.

### Proposition 6

Let $\delta = \delta (|H|n)>0$ be as in Conjecture $C_{|H|n}$. Let $\epsilon >0$. For any non-zero ideal ${\mathfrak {m}}\subset {\mathcal {O}}$, we have

21 $$\begin{aligned} \sum _{\begin{array}{c} {\mathfrak {N}}({\mathfrak {a}})\le x\\ ({\mathfrak {a}}, F) = 1, {\mathfrak {m}}\mid {\mathfrak {a}} \end{array}}s_{{\mathfrak {a}}} \ll x^{1-\frac{\delta }{54n|H|^2} + \epsilon }, \end{aligned}$$

where the implied constant depends only on *K* and $\epsilon$.

### Proposition 7

Let $\epsilon >0$. For any pair of sequences of complex numbers $\{v_{{\mathfrak {a}}}\}$ and $\{w_{{\mathfrak {b}}}\}$ indexed by non-zero ideals in ${\mathcal {O}}$ and satisfying $|v_{{\mathfrak {a}}}|, |v_{{\mathfrak {b}}}|\le 1$, we have

22 $$\begin{aligned} \sum _{\begin{array}{c} {\mathfrak {N}}({\mathfrak {a}})\le x \\ ({\mathfrak {a}}, F)=1 \end{array}}\sum _{\begin{array}{c} {\mathfrak {N}}({\mathfrak {b}})\le y\\ ({\mathfrak {b}}, F)=1 \end{array}}v_{{\mathfrak {a}}}w_{{\mathfrak {b}}}s_{{\mathfrak {a}}{\mathfrak {b}}} \ll \left( x^{-\frac{1}{6n}}+y^{-\frac{1}{6n}}\right) \left( xy\right) ^{1+\epsilon }, \end{aligned}$$

where the implied constant depends only on *K* and $\epsilon$.

### Proof of proposition 6

The proof is very similar to the proof of [6, (2.5), p. 7], so we will outline the additional arguments necessary to prove Proposition 6. For each non-zero ideal ${\mathfrak {a}}$, there exists a prime ideal ${\mathfrak {p}}\in {\mathcal {C}}$ such that ${\mathfrak {a}}{\mathfrak {p}}^2$ is principal. We can thus write

$$\begin{aligned} A_{{\mathfrak {m}}}(x) = \sum _{{\mathfrak {p}}\in {\mathcal {C}}}A_{{\mathfrak {m}}}(x; {\mathfrak {p}}), \end{aligned}$$

where

$$\begin{aligned} A_{{\mathfrak {m}}}(x; {\mathfrak {p}}) = \sum _{\begin{array}{c} {\mathfrak {N}}({\mathfrak {a}})\le x \\ ({\mathfrak {a}}, F) = 1,\ {\mathfrak {m}} \mid {\mathfrak {a}}\\ {\mathfrak {a}}{\mathfrak {p}}^2\text { is principal} \end{array}} s_{{\mathfrak {a}}}. \end{aligned}$$

Since ${\mathcal {C}}$ depends only on *K*, it now suffices to prove that

$$\begin{aligned} A_{{\mathfrak {m}}}(x; {\mathfrak {p}}) = \sum _{\begin{array}{c} {\mathfrak {N}}({\mathfrak {a}})\le x \\ ({\mathfrak {a}}, F) = 1,\ {\mathfrak {m}} \mid {\mathfrak {a}}\\ {\mathfrak {a}}{\mathfrak {p}}^2\text { is principal} \end{array}} s_{{\mathfrak {a}}} \ll x^{1-\frac{\delta }{54n|H|^2} + \epsilon } \end{aligned}$$

for each ${\mathfrak {p}}\in {\mathcal {C}}$, where the implied constant depends only on *K* and $\epsilon$. We now use the bijection (19), the formula (18), and the equivalence (20) to write

$$\begin{aligned} A_{{\mathfrak {m}}}(x; {\mathfrak {p}}) = \sum _{\begin{array}{c} \alpha _0\in {\mathcal {D}},\ {\mathfrak {N}}(\alpha _0)\le x{\mathfrak {N}}({\mathfrak {p}})^2 \\ (\alpha _0, F) = 1,\ \alpha _0\equiv 0\bmod [{\mathfrak {m}}, {\mathfrak {p}}^2] \\ \alpha _0^h\in A \end{array}} \prod _{\sigma \in H}\genfrac(){}{}{\alpha _0}{\sigma (\alpha _0)}, \end{aligned}$$

where $[{\mathfrak {m}}, {\mathfrak {p}}^2]$ denotes the least common multiple of ${\mathfrak {m}}$ and ${\mathfrak {p}}^2$. Again, since ${\mathcal {C}}$ and so also the norms $\{{\mathfrak {N}}({\mathfrak {p}})\}_{{\mathfrak {p}}\in {\mathcal {C}}}$ depend only on *K*, it suffices to prove that

23 $$\begin{aligned} A'_{{\mathfrak {m}}}(x) = \sum _{\begin{array}{c} \alpha \in {\mathcal {D}},\ {\mathfrak {N}}(\alpha )\le x \\ (\alpha , F) = 1,\ \alpha \equiv 0\bmod {\mathfrak {m}}\\ \alpha ^h\in A \end{array}} \prod _{\sigma \in H}\genfrac(){}{}{\alpha }{\sigma (\alpha )}\ll _{K, \epsilon } x^{1-\frac{\delta }{54n|H|^2} + \epsilon } \end{aligned}$$

uniformly for all non-zero ideals ${\mathfrak {m}}$. We have thus removed the issue of summing terms involving ${{\,\mathrm{spin}\,}}({\mathfrak {a}}, \sigma )$ for non-principal ideals ${\mathfrak {a}}$. It remains to handle the condition $\alpha ^h\in A$. To this end, we split the sum into congruence classes modulo *F*, and we emphasize that *F* is a multiple of 4. We get

$$\begin{aligned} A'_{{\mathfrak {m}}}(x) = \sum _{\begin{array}{c} \rho \bmod F \\ \rho \in \Omega _I(A) \end{array}}A'_{{\mathfrak {m}}}(x; \rho ), \end{aligned}$$

where

24 $$\begin{aligned} A'_{{\mathfrak {m}}}(x; \rho ) = \sum _{\begin{array}{c} \alpha \in {\mathcal {D}},\ {\mathfrak {N}}(\alpha )\le x \\ \alpha \equiv \rho \bmod F\\ \alpha \equiv 0\bmod {\mathfrak {m}} \end{array}}\prod _{\sigma \in H}\genfrac(){}{}{\alpha }{\sigma (\alpha )} \end{aligned}$$

and where $\Omega _I(A)$ is the set of congruence classes $\rho$ modulo *F* such that $(\rho , F) = 1$ and such that

$$\begin{aligned} \alpha \equiv \rho \bmod F\Longrightarrow \alpha ^h\in A. \end{aligned}$$

Note that $$|\Omega _I(A)|\le F^n \ll _{K} 1$$.

The sum $A'_{{\mathfrak {m}}}(x; \rho )$ in (24) is identical to the sum $A(x, \rho )$ in [6, (3.2), p. 9]. Hence, the bound for $A(x, \rho )$ proved in [6, Section 3] carries over to $A'_{{\mathfrak {m}}}(x; \rho )$, which, in conjunction with the fact that *F* depends only on *K*, implies the bound (23) and hence also Proposition 6.

### Proof of proposition 7

The proof is very similar to the proof of [6, (2.6), p. 7], so we will outline the additional arguments necessary to prove Proposition 7. Given $x, y>0$ and two sequences $v = \{v_{{\mathfrak {a}}}\}_{{\mathfrak {a}}}$ and $w = \{w_{{\mathfrak {b}}}\}_{{\mathfrak {b}}}$ of complex numbers bounded in modulus by 1, recall that we defined

25 $$\begin{aligned} B(x, y; v, w) = \sum _{\begin{array}{c} {\mathfrak {N}}({\mathfrak {a}})\le x\\ ({\mathfrak {a}}, F) = 1 \end{array}}\sum _{\begin{array}{c} {\mathfrak {N}}({\mathfrak {b}})\le y\\ ({\mathfrak {b}}, F) = 1 \end{array}}v_{{\mathfrak {a}}}w_{{\mathfrak {b}}}s_{{\mathfrak {a}}{\mathfrak {b}}}, \end{aligned}$$

and that our goal is to prove that

26 $$\begin{aligned} B(x, y; v, w) \ll _{K, \epsilon } \left( x^{-\frac{1}{6n}}+y^{-\frac{1}{6n}}\right) \left( xy\right) ^{1+\epsilon } \end{aligned}$$

for all $\epsilon >0$, uniformly in *v* and *w*. We can write

$$\begin{aligned} B(x, y; v, w) = \sum _{{\mathfrak {p}}_1\in {\mathcal {C}}}\sum _{{\mathfrak {p}}_2\in {\mathcal {C}}}B(x, y; v, w; {\mathfrak {p}}_1, {\mathfrak {p}}_2), \end{aligned}$$

where, for $({\mathfrak {p}}_1, {\mathfrak {p}}_2)\in {\mathcal {C}}\times {\mathcal {C}}$, we set

$$\begin{aligned} B(x, y; v, w; {\mathfrak {p}}_1, {\mathfrak {p}}_2) = \sum _{\begin{array}{c} {\mathfrak {N}}({\mathfrak {a}})\le x \\ ({\mathfrak {a}}, F) = 1\\ {\mathfrak {a}}{\mathfrak {p}}_1^2\text { is principal} \end{array}}\sum _{\begin{array}{c} {\mathfrak {N}}({\mathfrak {b}})\le y \\ ({\mathfrak {b}}, F) = 1\\ {\mathfrak {b}}{\mathfrak {p}}_2^2\text { is principal} \end{array}}v_{{\mathfrak {a}}}w_{{\mathfrak {b}}}s_{{\mathfrak {a}}{\mathfrak {b}}}. \end{aligned}$$

It suffices to prove the desired estimate for each of the $h^2$ sums $B(x, y; v, w; {\mathfrak {p}}_1, {\mathfrak {p}}_2)$. So fix $({\mathfrak {p}}_1, {\mathfrak {p}}_2)\in {\mathcal {C}}\times {\mathcal {C}}$. Writing $\pi _1$, $\pi _2$, $\alpha _0$, and $\beta _0$ for the totally positive generators of the principal ideals ${\mathfrak {p}}_1^h$, ${\mathfrak {p}}_2^h$, ${\mathfrak {a}}{\mathfrak {p}}_1^2$, and ${\mathfrak {b}}{\mathfrak {p}}_2^2$, respectively, we obtain in a similar way to (18) the formula

27 $$\begin{aligned} {{\,\mathrm{spin}\,}}({\mathfrak {a}}{\mathfrak {b}}, \sigma ) = \genfrac(){}{}{\alpha _0\beta _0}{\sigma (\alpha _0\beta _0)} = \genfrac(){}{}{\alpha _0}{\sigma (\alpha _0)}\genfrac(){}{}{\beta _0}{\sigma (\beta _0)}\genfrac(){}{}{\alpha _0}{\sigma (\beta _0)\sigma ^{-1}(\beta _0)}. \end{aligned}$$

Using the bijection (19), the formula (27), and the equivalence (20), we deduce that

28 $$\begin{aligned} B(x, y; v, w; {\mathfrak {p}}_1, {\mathfrak {p}}_2) = \sum _{\begin{array}{c} \alpha _0\in {\mathcal {D}}\\ {\mathfrak {N}}(\alpha _0)\le x{\mathfrak {N}}({\mathfrak {p}}_1)^2 \\ (\alpha _0, F) = 1 \\ \alpha _0\equiv 0\bmod {\mathfrak {p}}_1^2 \end{array}}\sum _{\begin{array}{c} \beta _0\in {\mathcal {D}}\\ {\mathfrak {N}}(\beta _0)\le y{\mathfrak {N}}({\mathfrak {p}}_2)^2 \\ (\beta _0, F) = 1\\ \beta _0 \equiv 0\bmod {\mathfrak {p}}_2^2 \\ (\alpha _0\beta _0)^h\in A \end{array}}v'_{\alpha _0}w'_{\beta _0}\phi (\alpha _0, \beta _0), \end{aligned}$$

where

$$\begin{aligned} v'_{\alpha _0} = v_{(\alpha _0)/{\mathfrak {p}}_1^2}\prod _{\sigma \in H}\genfrac(){}{}{\alpha _0}{\sigma (\alpha _0)}\quad \text {and}\quad w'_{\beta _0} = w_{(\beta _0)/{\mathfrak {p}}_2^2}\prod _{\sigma \in H}\genfrac(){}{}{\beta _0}{\sigma (\beta _0)} \end{aligned}$$

and where $\phi (\cdot ,\cdot )$ is the same function as the one defined in [6, p. 19], i.e.,

$$\begin{aligned} \phi (\alpha _0, \beta _0) = \prod _{\sigma \in H}\genfrac(){}{}{\alpha _0}{\sigma (\beta _0)\sigma ^{-1}(\beta _0)}. \end{aligned}$$

We further split the sum $B(x, y; v, w; {\mathfrak {p}}_1, {\mathfrak {p}}_2)$ into congruence classes modulo *F*. As *F* is divisible by 4, this will have the effect of separating the variables $\alpha _0$ and $\beta _0$ in the condition $(\alpha _0\beta _0)^h\in A$. We have

$$\begin{aligned} B(x, y; v, w; {\mathfrak {p}}_1, {\mathfrak {p}}_2) = \sum _{\rho _1\bmod F}\sum _{\begin{array}{c} \rho _2\bmod F \\ (\rho _1, \rho _2)\in \Omega _{II}(A) \end{array}}B(x, y; v, w; {\mathfrak {p}}_1, {\mathfrak {p}}_2; \rho _1, \rho _2), \end{aligned}$$

where

$$\begin{aligned} B(x, y; v, w; {\mathfrak {p}}_1, {\mathfrak {p}}_2; \rho _1, \rho _2) = \sum _{\begin{array}{c} \alpha _0\in {\mathcal {D}}\\ {\mathfrak {N}}(\alpha _0)\le x{\mathfrak {N}}({\mathfrak {p}}_1)^2 \\ \alpha _0\equiv \rho _1\bmod F \end{array}}\sum _{\begin{array}{c} \beta _0\in {\mathcal {D}}\\ {\mathfrak {N}}(\beta _0)\le y{\mathfrak {N}}({\mathfrak {p}}_2)^2 \\ \beta _0 \equiv \rho _2\bmod F \end{array}}v''_{\alpha _0}w''_{\beta _0}\phi (\alpha _0, \beta _0). \end{aligned}$$

Here

$$\begin{aligned} v''_{\alpha _0} = \mathbf{1 }(\alpha _0\equiv 0\bmod {\mathfrak {p}}_1^2) \cdot v'_{\alpha _0} \end{aligned}$$

and

$$\begin{aligned} w''_{\beta } = \mathbf{1 }(\beta _0\equiv 0\bmod {\mathfrak {p}}_2^2)\cdot w'_{\beta _0}, \end{aligned}$$

where $\mathbf{1 }(P)$ is the indicator function of a property *P*, and $\Omega _{II}(A)$ is the set of $(\rho _1, \rho _2)\in ({\mathcal {O}}/(F))^{\times }\times ({\mathcal {O}}/(F))^{\times }$ such that

$$\begin{aligned} \alpha _0\equiv \rho _1\bmod F\text { and }\beta _0\equiv \rho _2\bmod F\Longrightarrow (\alpha _0\beta _0)^h\in A. \end{aligned}$$

Note that $$|\Omega _{II}(A)|\le F^2$$.

The sum $B(x, y; v, w; {\mathfrak {p}}_1, {\mathfrak {p}}_2; \rho _1, \rho _2)$ has the same shape as the sum $B_i(x, y; \alpha _0, \beta _0)$ in [6, p. 19], and so the bound [6, (4.5), p. 19] implies that

$$\begin{aligned} B(x, y; v, w; {\mathfrak {p}}_1, {\mathfrak {p}}_2; \rho _1, \rho _2) \ll _{K, \epsilon } \left( x^{-\frac{1}{6n}}+y^{-\frac{1}{6n}}\right) \left( xy\right) ^{1+\epsilon }. \end{aligned}$$

This finishes the proof of Proposition 7 and hence also of Theorem 6.

## Proof of main results

We now prove Theorem 1.

### Proof

By Theorem 4, for each sign $\mu \in \{\pm \}$, $d(R_\mu |S_\mu )= \#\ker (\star _\mu )/2^{(n-1)}$. Then $d(R_\mu |S_\mu )=s_\mu /2^{(n-1)}$ by Proposition 5. By Theorem 5, $d(F_\mu |R_\mu ) = 2^{-(n-1)/2}$. Therefore

$$\begin{aligned} d(F_\mu |S_\mu ) =d(F_\mu |R_\mu )d(R_\mu |S_\mu )= \frac{s_\mu }{2^{3(n-1)/2}}. \end{aligned}$$

Since $$d(F|S)=d(F_+|S_+)d(S_+|S)+d(F_-|S_-)d(S_-|S)$$, and $$d(S_\mu |S)=1/2$$,

$$\begin{aligned} d(F|S)=\frac{s_++s_-}{2^{(3n-1)/2}}. \end{aligned}$$

$$\square $$

Theorem 1 settles Conjecture 1.1 in [9]. This conjecture was originally stated for number fields *K* which in addition to satisfying properties (C1)-(C4), were also assumed to have prime degree. While as originally stated, this assumption is necessary, it is artificial here. In [9], $m_K$ is defined as the number of non-trivial ${{\,\mathrm{Gal}\,}}(K/{\mathbb {Q}})$-orbits of ${\mathbf {M}}_4$ with representative $\alpha \in {\mathcal {O}}$ such that $(\alpha ,\alpha ^\sigma )_2=1$. Let *s* denote the number of *elements* of ${\mathbf {M}}_4$ with representative $\alpha \in {\mathcal {O}}$ such that $(\alpha ,\alpha ^\sigma )_2=1$. When *n* is prime, $s=m_Kn+1$.

Let *E* denote the set of rational primes *p* such that for ${\mathfrak {p}}$ a prime of *K* above *p*, ${{\,\mathrm{spin}\,}}({\mathfrak {p}},\sigma )=1$ for all non-trivial $\sigma \in {{\,\mathrm{Gal}\,}}(K/{\mathbb {Q}})$. For a fixed sign $\mu \in \{\pm \}$, let $E_\mu$ denote the set of primes of *E* congruent to $\mu 1\bmod 4$.

Conjecture 1.1 in [9] made two assertions, one regarding the density *d*(*E*|*S*) of such primes restricted to those splitting completely in $K/{\mathbb {Q}}$ and one regarding the overall density *d*(*E*) of such primes. The assertion regarding the restricted density is correct and the assertion regarding the overall density is slightly off due to a very simple error in the case in which *p* is not assumed to split completely in $K/{\mathbb {Q}}$. Theorem 7 proves conditionally a slight modification of Conjecture 1.1 in [9]

### Theorem 7

[9] Let *K* be a number field with prime degree satisfying properties (C1)-(C4). Assume Conjecture $C_{\eta }$ holds for $\eta = \frac{2}{n(n-1)}$ with $n=[K:{\mathbb {Q}}]$. Then

$$\begin{aligned} d(E|S) = \frac{s}{2^{(3n-1)/2}}, \quad d(E) = \frac{s}{n2^{(3n-1)/2}}, \end{aligned}$$

$$\begin{aligned} d(E_\mu |S_\mu ) = \frac{s_\mu }{2^{3(n-1)/2}}, \quad \text {and} \quad d(E_\mu ) = \frac{s_\mu }{n2^{(3n-1)/2}}. \end{aligned}$$

When *n* is prime, $s=m_Kn+1$.

### Proof

If ${\mathfrak {p}}$ is a prime of *K* that does not split completely in $K/{\mathbb {Q}}$, then for some non-trivial $\sigma \in {{\,\mathrm{Gal}\,}}(K/{\mathbb {Q}})$, ${\mathfrak {p}}^\sigma ={\mathfrak {p}}$ so ${{\,\mathrm{spin}\,}}({\mathfrak {p}},\sigma )=0$. Therefore $E\subseteq S$ so this *E* is exactly the *F* studied in Theorem 1 and $E_\mu =F_\mu$.

Then $d(E) = d(F) = d(F|S)d(S)$ and $d(E_\mu ) = d(F_\mu ) = d(F_\mu |S_\mu )d(S_\mu )$. Since $d(S)=1/n$ by the Chebotarev Density Theorem and $d(S_\mu ) = 1/(2n)$, the result follows from Theorem 1. $\square$

In Theorem 2, *K* satisfies (C1), (C2), and (C4) directly from the assumptions. When *K* is a cyclic cubic number field with odd class number, by [1, Theorem V] all signatures are represented by units so condition (C3) is satisfied by Lemma 1 because *h* is odd. It is a consequence of the classical Burgess’s inequality [2] that Conjecture $C_{\eta }$ is true for $\eta = \frac{2}{3(3-1)} = \frac{1}{3}$, as is shown in Section 9 of [5]. Therefore Theorem 2 follows from Theorem 1 and is unconditional.
